# Supplementary material for: Systematic comparison of family history and polygenic risk across 24 common diseases
Source: Am J Hum Genet. 2022 Nov 7;109(12):2152–62. doi: 10.1016/j.ajhg.2022.10.009 (PMC9748261; doi:10.1016/j.ajhg.2022.10.009)
Supplement: Document S1. Figures S1–S10 [file mmc1.pdf]

The American Journal of Human Genetics, Volume *109*

Supplemental information

Systematic comparison of family history and polygenic risk across 24 common diseases

Nina Mars, Joni V. Lindbohm, Pietro della Briotta Parolo, Elisabeth Widén, Jaakko Kaprio, Aarno Palotie, FinnGen, and Samuli Ripatti

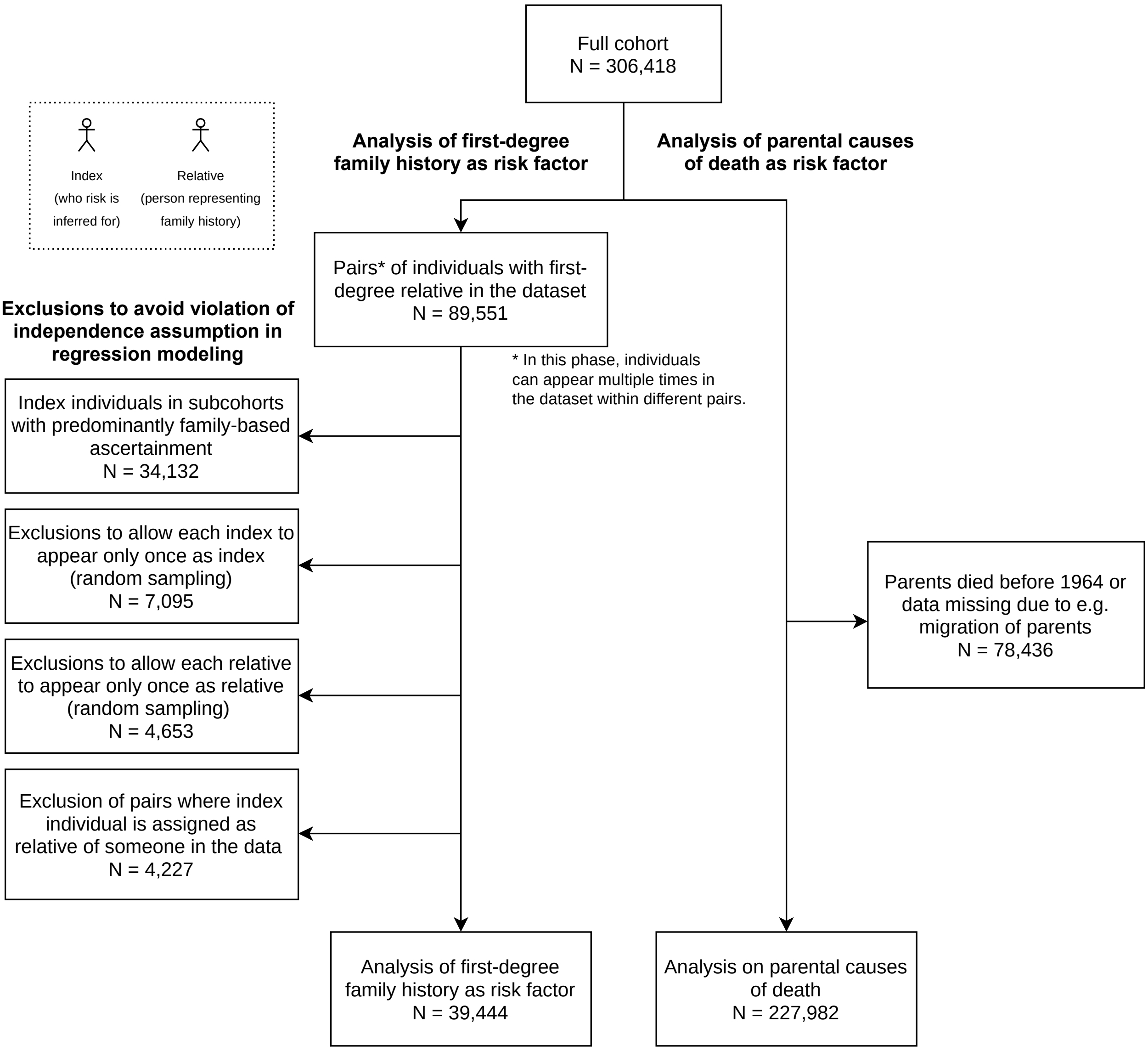

Figure S1. Study flowchart. The flowchart describes generation of datasets used for the definitions first-degree family history and parental cause of death. Having inferred kinship, the initial data structure allows individuals to appear multiple times in the dataset within different pairs. The main processing steps after this involved random exclusions, to individuals appearing multiple times on either side of the regression equation, which would violate the assumption of independence of observations. Similar steps were performed for second-degree family history as for first degree family history, starting from 118,992 pairs of individuals with a second-degree relative in the dataset and resulting in 47,154 individuals for analysis of second-degree family history as a risk factor. For breast cancer, we studied only pairs of women (15,281 individuals, parent-offspring relationship in 7,770; full-sibling relationship in 7,511). For prostate cancer, we studied only pairs of men (9,473 individuals, parent-offspring relationship in 3,932; full-sibling relationship in 5,541).

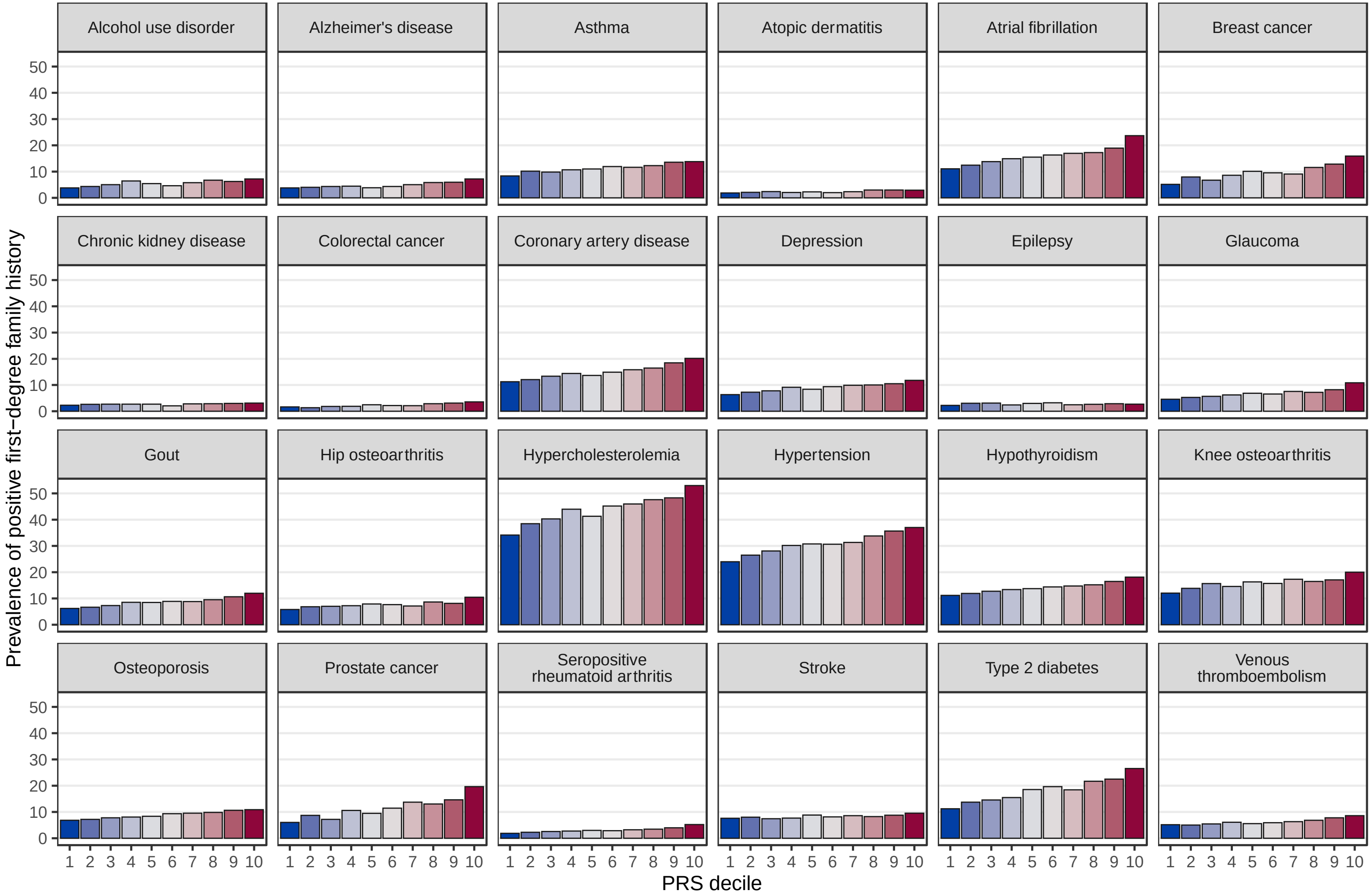

Figure S2. Prevalence of first-degree family history (FH_{1st}) by deciles of polygenic risk score (PRS). Total N = 39,444, N = 15,281 for breast cancer, N = 9,473 for prostate cancer.

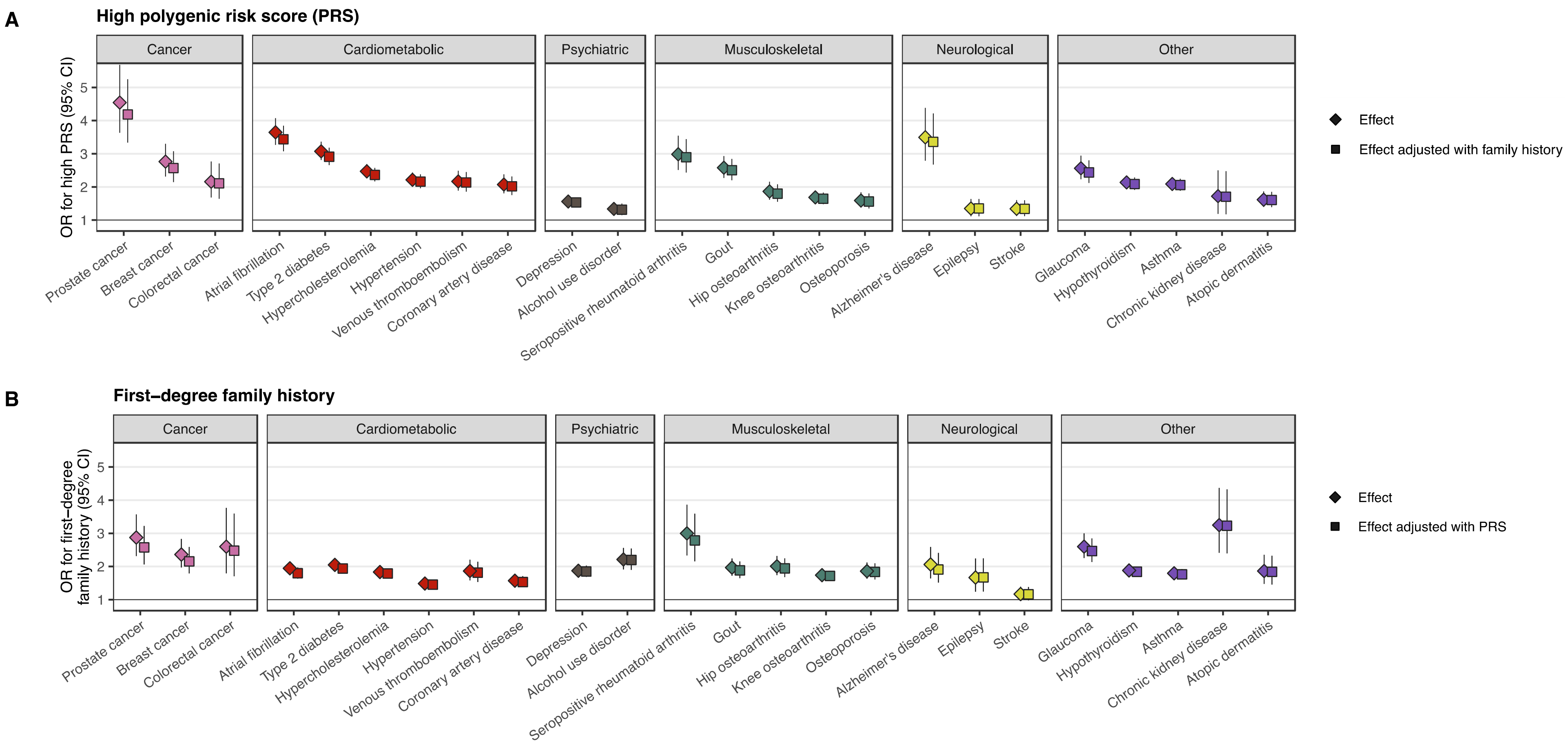

Figure S3. Cross-adjustment effects for family history and polygenic risk score (PRS) with PRS categorized. The impact of adjusting the PRS effect with first-degree family history **(**FH$_{1st,}$ **panel A)** and vice versa **(panel B)**. The diamonds represent the unadjusted effects and the squares the adjusted effects. The PRS effect size compares individuals in the top decile of the PRS distribution to the rest. Total N = 39,444, N = 15,281 for breast cancer, N = 9,473 for prostate cancer. Odds ratios (OR) were obtained from logistic regression models adjusted for sex (except for breast and prostate cancer), birth year, genotyping array, cohort, and the first ten genetic principal components of ancestry.

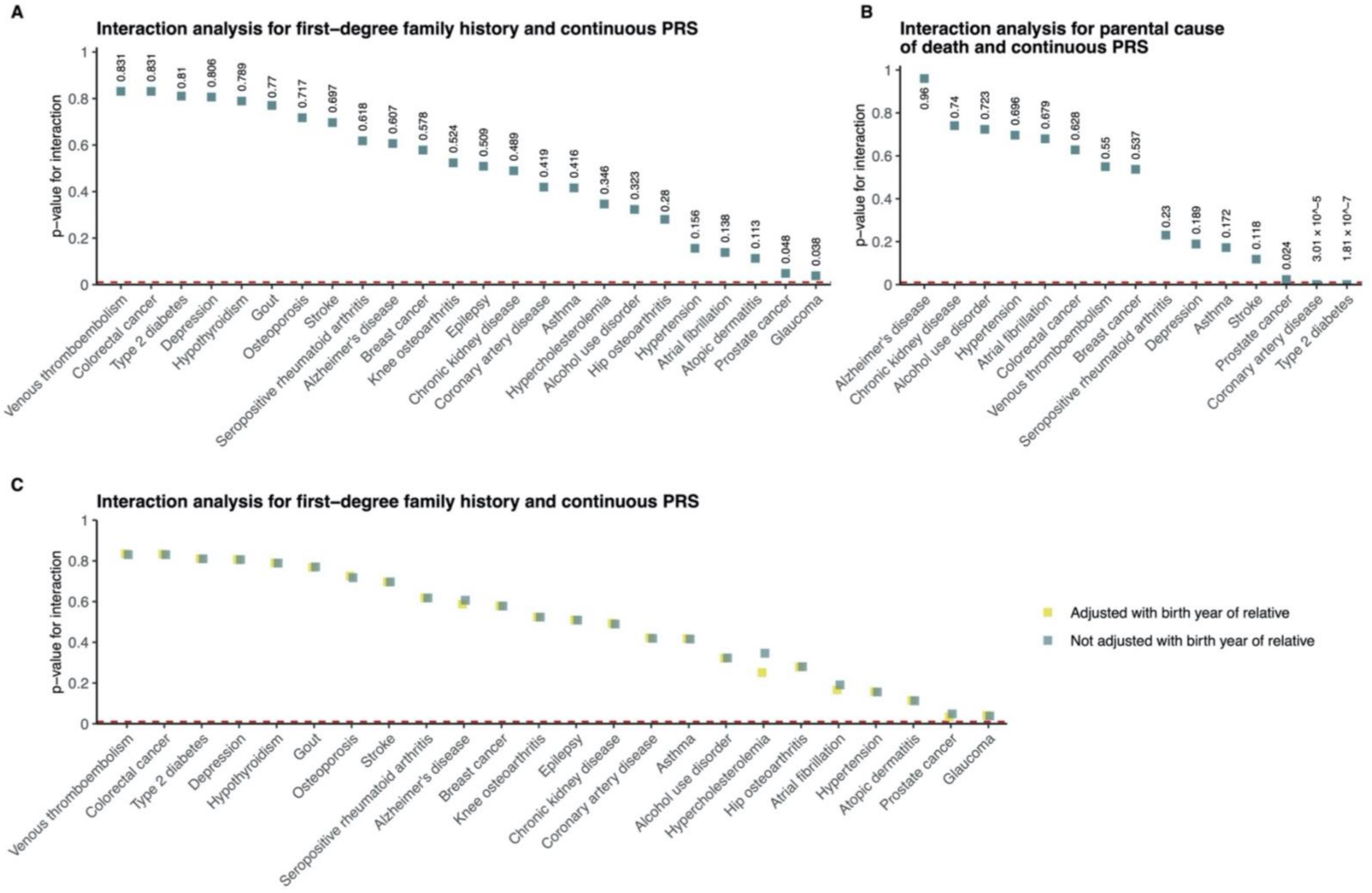

Figure S4. Interaction analysis. Interaction analysis between first-degree family history (FH_{1st}, **panel A**) or parental causes of death (FH_P, **panel B**) and respective polygenic risk scores (PRS), displaying the p-value for the interaction term on the y-axis. **Panel C** shows the results of **panel B** adjusting also for birth year of the relative. The PRSs were scaled to zero mean and unit variance and handled as continuous variables in the interaction analysis. Statistical significance set at a p-value threshold of 0.0013 (Bonferroni-correction for 24+15 tests) represented by the red line. We did not observe systematic evidence of interactions. Total N = 39,444, N = 15,281 for breast cancer, N = 9,473 for prostate cancer. The logistic regression models were adjusted for sex (except for breast and prostate cancer), birth year, genotyping array, cohort, and the first ten genetic principal components of ancestry.

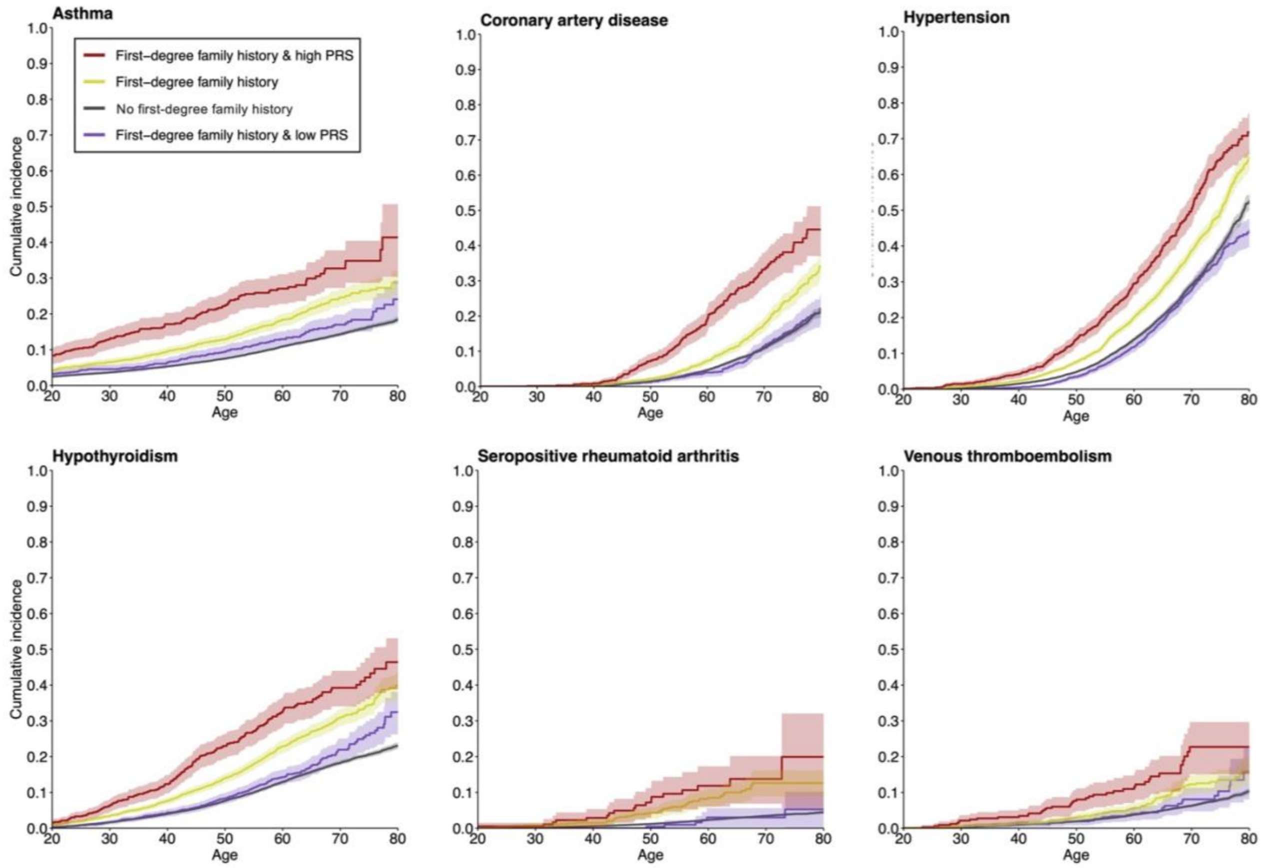

Figure S5. The impact of polygenic risk on disease risk in individuals with positive family history. Figure 6 showed results for the five diseases with the largest effect sizes for PRS, and for breast and prostate cancer, with the rest of the diseases fulfilling the criteria of an OR >2 for high PRS (Table S7) and over 10 cases in each subgroup shown here. The survival curves show cumulative incidences for individuals with positive first-degree family (FH_{1st}), stratified by level of polygenic risk score (PRS). High PRS was defined as top decile of the PRS distribution and low PRS as the bottom tertile of the PRS distribution. Total N = 39,444, N = 15,281 for breast cancer, N = 9,473 for prostate cancer.

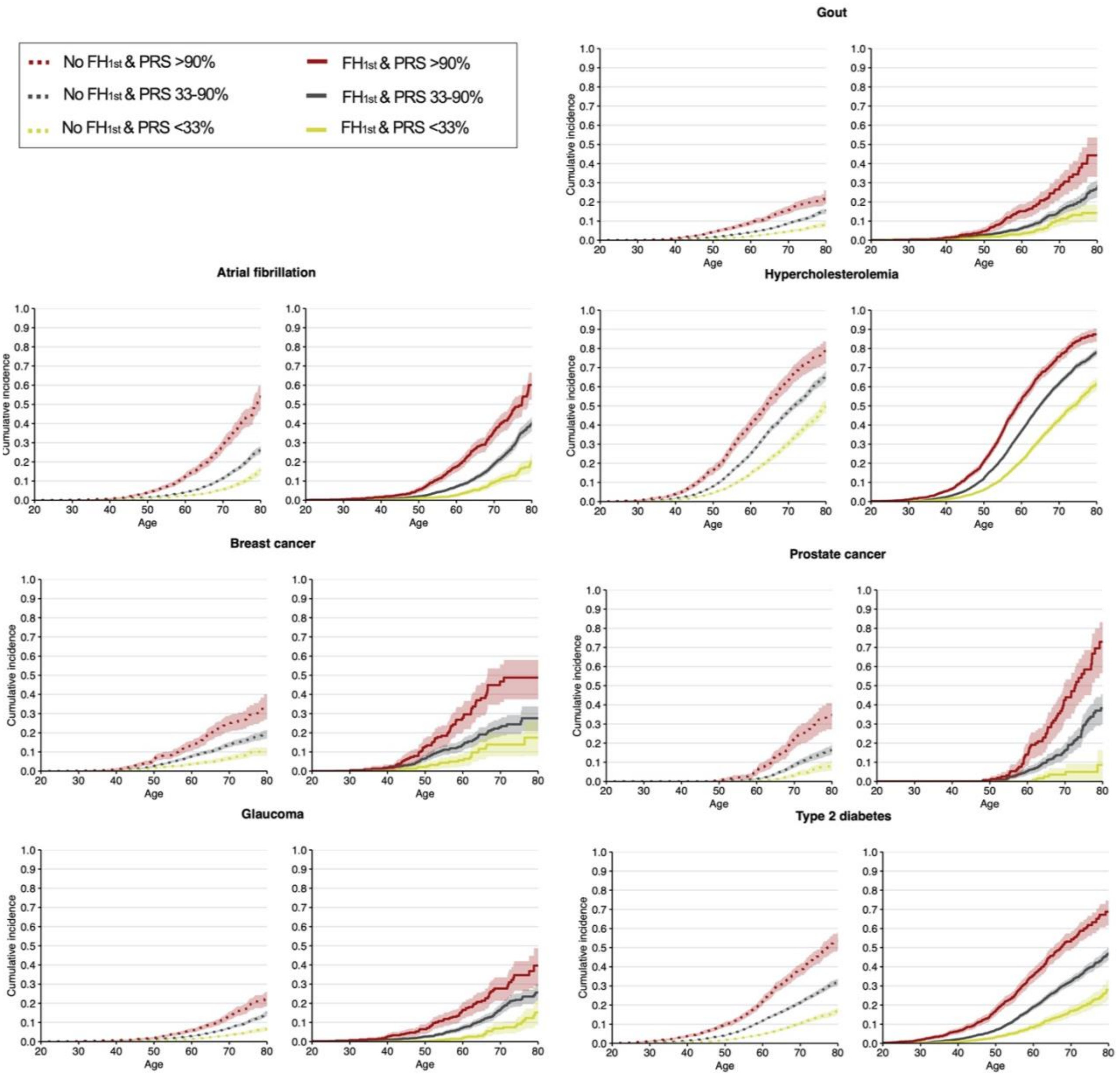

Figure S6. Polygenic risk scores (PRS) stratified by negative and positive family history. Impact of the level of PRS on cumulative incidence of disease in individuals with negative (dashed lines) and positive (solid line) first-degree family (FH_{1st}). High PRS was defined as top decile of the PRS distribution and low PRS as the bottom tertile of the PRS distribution. A low PRS compensated for the impact of positive FH_{1st}, whereas individuals with a combination of high PRS and positive FH_{1st} had a particularly high risk. The figure shows results for the five diseases with the largest effect sizes for PRS, and for breast and prostate cancer. Total N = 39,444, N = 15,281 for breast cancer, N = 9,473 for prostate cancer.

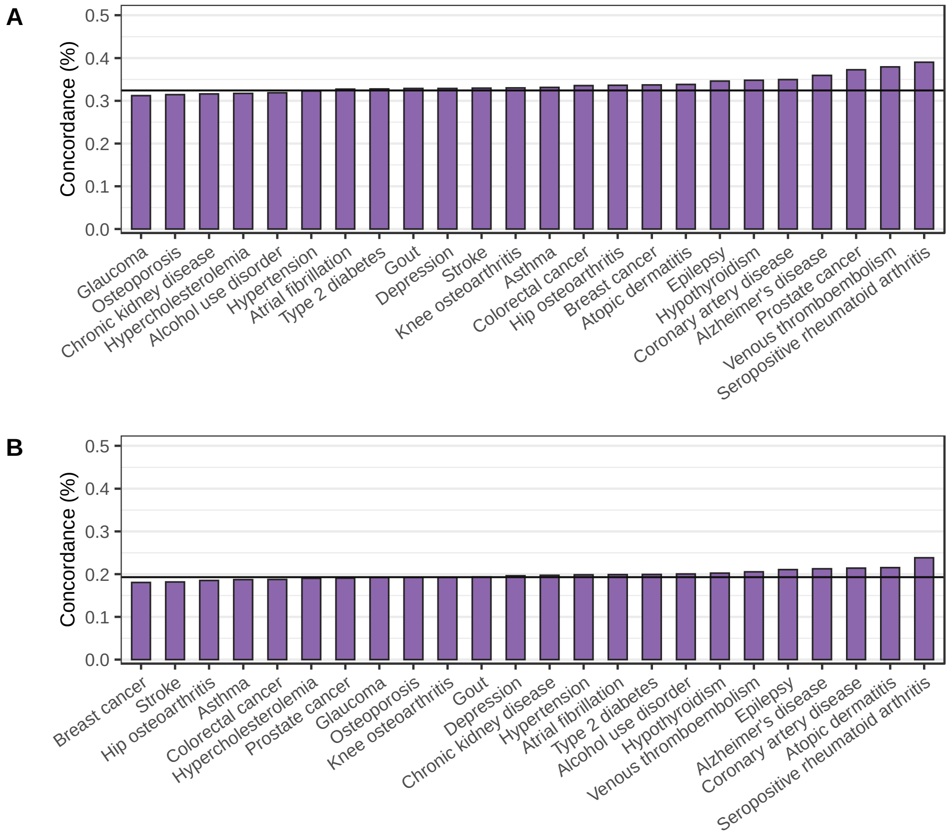

Figure S7. Polygenic risk score (PRS) concordance. Concordance of a high PRS (defined as top 10% of the distribution) among first-degree relatives (**panel A**) and among second-degree relatives (**panel B**). The horizontal lines denote the theoretically derived concordance estimates of 32.4% (**panel A**) and 19.3% (**panel B**) calculated based on reference 47 for first-degree relatives using a high PRS defined as the top 10% of the distribution. Sample sizes: panel A total N = 39,444, N = 15,281 for breast cancer, N = 9,473 for prostate cancer; panel B second -degree family history total N = 47,154, N = 18,973 for breast cancer, N = 12,355 for prostate cancer.

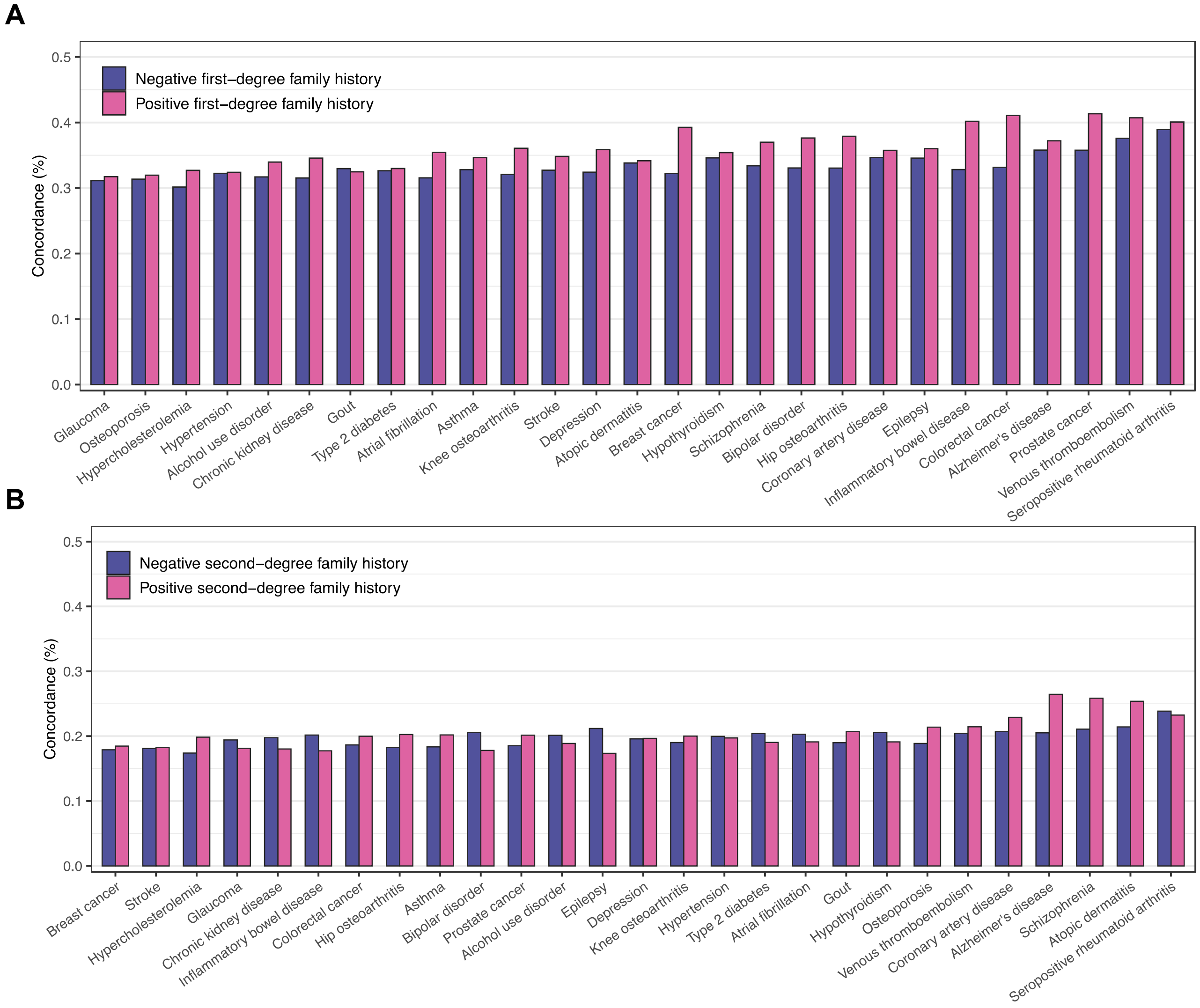

Figure S8. Polygenic risk score (PRS) concordance by family history status. Concordance of a high PRS (defined as top 10% of the distribution) among first-degree relatives (**panel A**) and among second-degree relatives (**panel B**) stratifying by the respective family history status. Sample sizes: panel A total N = 39,444, N = 15,281 for breast cancer, N = 9,473 for prostate cancer; panel B second -degree family history total N = 47,154, N = 18,973 for breast cancer, N = 12,355 for prostate cancer.

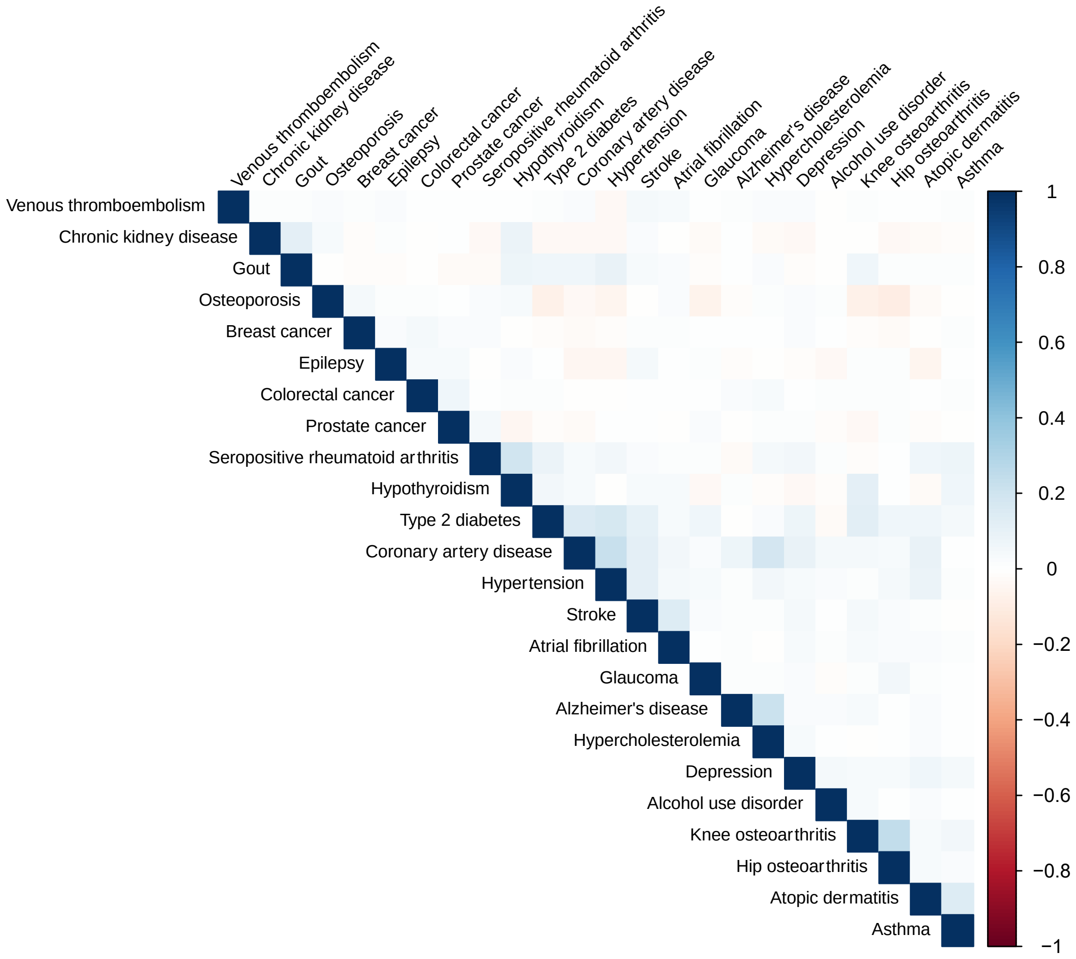

Figure S9. Polygenic risk scores (PRS) correlations .Pearson correlation of the 24 disease-specific PRSs assessed on the continuous scale.

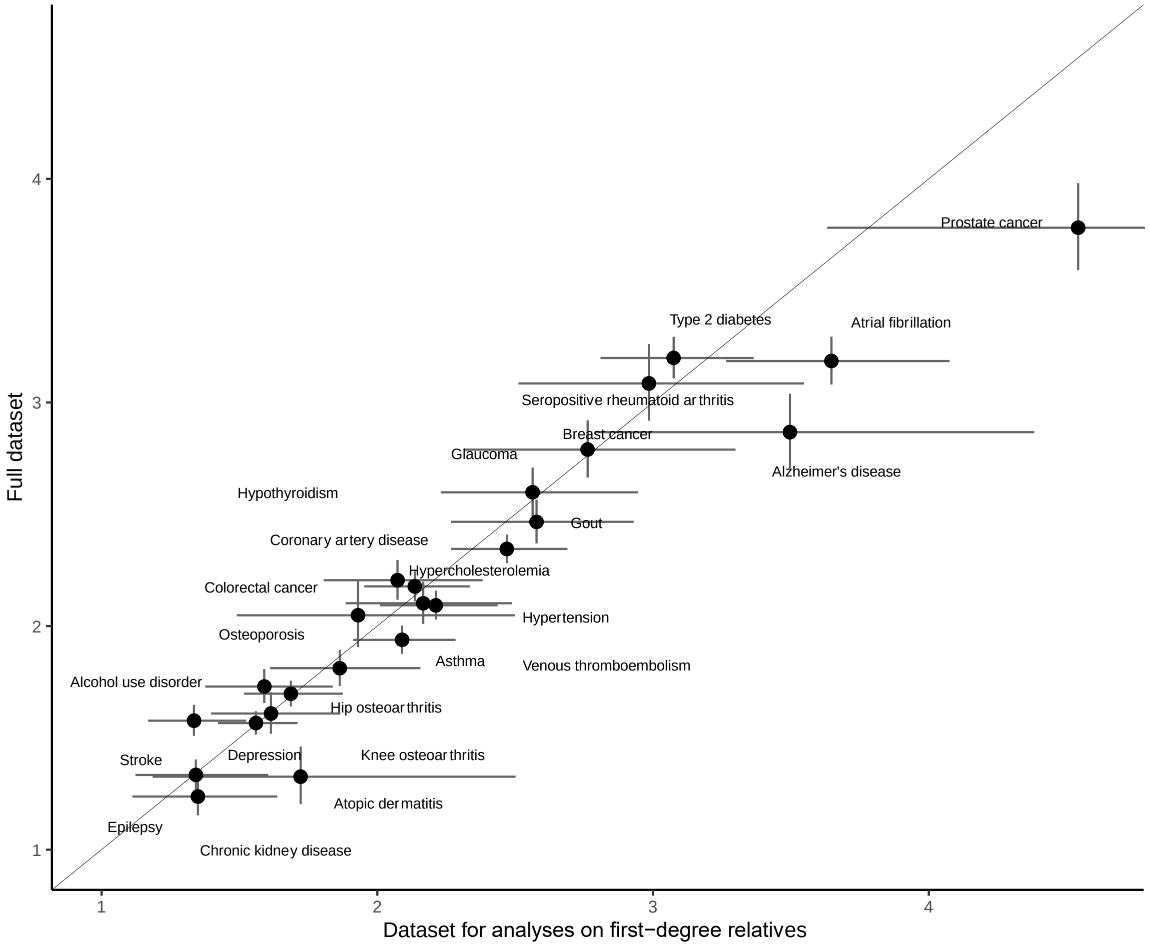

Figure S10. Effect size comparison for the full FinnGen dataset and the dataset used for analyses on first-degree relatives. Effect sizes for a high polygenic risk score (PRS; defined as top 10% of the distribution), comparing individuals in the full data (N = 306,418) on the y axis and the dataset used for analyses on first-degree relatives (N = 39,444) on the x axis. The PRS effect sizes were similar in both.

Supplemental Material and Methods

Identification of individuals with diseases included harmonization of diagnoses according to different revisions of International Statistical Classification of Diseases (ICD-8/9/10) (**Table S3**). Registries used include the hospital discharge registry (available from 1968-), the Finnish Cancer Registry (1953-), causes of death registry (1964-), and the medication reimbursement and medication purchases registries (1964- and 1995-), both administered by the Social Insurance Institute of Finland. Age at disease onset was defined as the age at the first healthcare contact with the disease. Age at death from the causes of death registry as recorded on the death certificate and checked by Statistics Finland. Parental cause of death (FH_P) was defined as at least one parent having the disease as a cause of death. Prostate cancer was studied only in men and breast cancer only in women.

Genotyping and imputation

FinnGen samples were genotyped with Illumina and Affymetrix arrays (Illumina Inc., San Diego, and Thermo Fisher Scientific, Santa Clara, CA, USA), and genotype calls were made with the GenCall or zCall (for Illumina) and the AxiomGT1 algorithm for Affymetrix data. Individuals with ambiguous gender (markers on the X chromosome not matching the sex denoted by the personal identification number), high genotype missingness (>5%), excess heterozygosity (+-4SD) and non-Finnish ancestry were excluded, as well as all variants with high missingness (>2%), low Hardy–Weinberg equilibrium p-value (<1e-6) and minor allele count (MAC < 3). Array data pre-phasing was carried out with Eagle 2.3.5[1] with the number of conditioning haplotypes set to 20,000. Genotype imputation was done using the population-specific SISu v3 imputation reference with 3,775 high-coverage (25-30x) whole-genome sequences in Finns, described in detail at https://doi.org/10.17504/protocols.io.xbgfijw.

Polygenic risk scores

Details on the GWASs used as the input for the PRSs are available in **Table S2**. Using these as priors, we applied the PRS-CS-auto algorithm to infer posterior effect sizes for the variants for PRS calculation. PRS-CS-auto learns the model's global scaling parameter ϕ from the data, performing well with large datasets.[2] The PRS-CS pipeline in FinnGen is described at https://github.com/FINNGEN/CS-PRS-pipeline. The PRSs for autosomes were calculated using PLINK v2.00a2.3LM, by calculating the weighted sum of risk alleles for each variant with the parameter *--score* applied on a genotype file (all chromosomes combined) which had been filtered to 1,194,526 HapMap3 variants, as recommended for PRS-CS. The mean number of variants included in the PRSs was 1,059,217. We observed very little correlation between the PRSs (**Figure S9**). To avoid overfitting of effects, the individuals potentially overlapping with the GWASs were excluded from all analyses based on genotyping array and cohort information (**Table S1, Table S2**). As we are unable to identify the exact individuals overlapping, we chose to use this conservative exclusion approach.

Inferring relatedness

To define first-degree family history (FH_{1st}), we inferred first-degree relatedness from genotypes based on 173,907 independent linkage disequilibrium (LD)-pruned common variants 9 (PLINK parameters --snps-only --chr 1-22 --max-alleles 2 --maf 0.01 --indep-pairwise 500.0 50.0 0.15). The LD pruning was done using variants with INFO >0.9. Using KING v2.2.4[3], pairs of first-degree relatives were identified with a kinship coefficient between 0.177 and 0.354, and pairs of second-degree relatives with a kinship coefficient between 0.0884 and 0.177. To avoid individuals appearing multiple times on either side of the regression equation which would violate the assumption of independence of observations, we performed several steps of random exclusions and exclusions of cohorts predominantly family-based ascertainment (**Figure S1**). We inferred the risk for the individual born later and the individual born earlier was chosen as the relative (random choice for dizygotic twins). The PRS effect sizes were similar in the full data and in those with a first-degree relative in the dataset (**Figure S10**). In a previous study,[4] we performed a comparison of self-reported first-degree family history and PRSs for coronary artery disease (CAD) and type 2 diabetes (T2D) in the Finnish FINRISK cohort. The prevalence of first-degree family history was slightly higher in FINRISK (22.2% for CAD and 25.8% in T2D) than the prevalences for FH_{1st} observed here (15.1% for CAD and 18.6% for T2D; **Table S4**), but the dynamics of family history and PRS were highly similar.

Parental causes of death

Parental causes of death (FH_P) was available for participants regardless of whether their parents are included in FinnGen. FH_P was obtained through the Death Registry, which has nationwide coverage. Information on the parents' sex, age at death, and causes of death (immediate, contributing, and underlying causes of death) was available for FinnGen participants. We studied FH_P to obtain a robust and complementary source of data

that does not have the same limitations as the approach used for FH_{1st} and FH_{2nd}, which required that at least one relative is included in FinnGen.

Ethics statement

Individuals and controls in FinnGen provided informed consent for biobank research, based on the Finnish Biobank Act. Alternatively, separate research cohorts, collected prior the Finnish Biobank Act came into effect (in September 2013) and start of FinnGen (August 2017), were collected based on study-specific consents and later transferred to the Finnish biobanks after approval by Fimea (Finnish Medicines Agency), the National Supervisory Authority for Welfare and Health. Recruitment protocols followed the biobank protocols approved by Fimea. The Coordinating Ethics Committee of the Hospital District of Helsinki and Uusimaa (HUS) statement number for the FinnGen study is Nr HUS/990/2017.

The FinnGen study is approved by Finnish Institute for Health and Welfare (permit numbers: THL/2031/6.02.00/2017, THL/1101/5.05.00/2017, THL/341/6.02.00/2018, THL/2222/6.02.00/ 2018, THL/283/6.02.00/2019, THL/1721/5.05.00/2019, THL/1524/5.05.00/2020, and THL/2364/ 14.02/2020), Digital and population data service agency (permit numbers: VRK43431/2017-3, VRK/6909/2018-3, VRK/4415/2019-3), the Social Insurance Institution (permit numbers: KELA 58/522/2017, KELA 131/522/2018, KELA 70/522/2019, KELA 98/522/2019, KELA 138/522/2019, KELA 2/522/2020, KELA 16/522/2020, Findata THL/2364/14.02/2020 and Statistics Finland (permit numbers: TK-53-1041-17 and TK/143/07.03.00/2020 (earlier TK-53-90-20).

The Biobank Access Decisions for FinnGen samples and data utilized in FinnGen Data Freeze 7 include: THL Biobank BB2017_55, BB2017_111, BB2018_19, BB_2018_34, BB_2018_67, BB2018_71, BB2019_7, BB2019_8, BB2019_26, BB2020_1, Finnish Red Cross Blood Service Biobank 7.12.2017, Helsinki Biobank HUS/359/2017, Auria Biobank AB17-5154 and amendment #1 (August 17 2020), Biobank Borealis of Northern Finland_2017_1013, Biobank of Eastern Finland 1186/2018 and amendment 22 § /2020, Finnish Clinical Biobank Tampere MH0004 and amendments (21.02.2020 & 06.10.2020), Central Finland Biobank 1-2017, and Terveystalo Biobank STB 2018001.

FinnGen acknowledgements

We would like to thank Mervi Aavikko and Risto Kajanne for management assistance. The FinnGen project is funded by two grants from Business Finland (HUS 4685/31/2016 and UH 4386/31/2016) and the following industry partners: AbbVie Inc., AstraZeneca UK Ltd, Biogen MA Inc., Bristol Myers Squibb, Genentech Inc., Merck Sharp & Dohme Corp, Pfizer Inc., GlaxoSmithKline Intellectual Property Development Ltd., Sanofi US Services Inc., Maze Therapeutics Inc., Janssen Biotech Inc, and Novartis Pharma AG. Following biobanks are acknowledged for delivering biobank samples to FinnGen: Auria Biobank (www.auria.fi/biopankki), THL Biobank (www.thl.fi/biobank), Helsinki Biobank (www.helsinginbiopankki.fi), Biobank Borealis of Northern Finland (https://www.ppshp.fi/Tutkimus-ja-opetus/Biopankki/Pages/Biobank-Borealis-briefly-in-English.aspx), Finnish Clinical Biobank Tampere (www.tays.fi/en-US/Research_and_development/Finnish_Clinical_Biobank_Tampere), Biobank of Eastern Finland (www.ita-suomenbiopankki.fi/en), Central Finland Biobank (www.ksshp.fi/fi-FI/Potilaalle/Biopankki), Finnish Red Cross Blood Service Biobank (www.veripalvelu.fi/verenluovutus/biopankkitoiminta) and Terveystalo Biobank (www.terveystalo.com/fi/Yritystietoa/Terveystalo-Biopankki/Biopankki/). All Finnish Biobanks are members of BBMRI.fi infrastructure (www.bbmri.fi) and FINBB biobank cooperative (https://finbb.fi/) is the coordinator of the BBMRI-ERIC operations in Finland covering all Finnish biobanks.

Supplemental references

1. Loh, P.R., Danecek, P., Palamara, P.F., Fuchsberger, C., Reshef, Y.A., Finucane, H.K., Schoenherr, S., Forer, L., McCarthy, S., Abecasis, G.R., et al. (2016). Reference-based phasing using the Haplotype Reference Consortium panel. Nat. Genet. *48*, 1443-1448.
2. Ge, T., Chen, C.Y., Ni, Y., Feng, Y.A., and Smoller, J.W. (2019). Polygenic prediction via Bayesian regression and continuous shrinkage priors. Nat Commun *10*, 1776.
3. Manichaikul, A., Mychaleckyj, J.C., Rich, S.S., Daly, K., Sale, M., and Chen, W.M. (2010). Robust relationship inference in genome-wide association studies. Bioinformatics *26*, 2867-2873.
4. Mars, N., Koskela, J.T., Ripatti, P., Kiiskinen, T.T.J., Havulinna, A.S., Lindbohm, J.V., Ahola-Olli, A., Kurki, M., Karjalainen, J., Palta, P., et al. (2020). Polygenic and clinical risk scores and their impact on age at onset and prediction of cardiometabolic diseases and common cancers. Nat. Med. *26*, 549-557.
