# Supplementary material for: Systematic comparison of family history and polygenic risk across 24 common diseases
Source: Am J Hum Genet. 2022 Nov 7;109(12):2152–62. doi: 10.1016/j.ajhg.2022.10.009 (PMC9748261; doi:10.1016/j.ajhg.2022.10.009)
Supplement: Document S2. Article plus supplemental information [file mmc3.pdf]

Systematic comparison of family history and polygenic risk across 24 common diseases

Graphical abstract

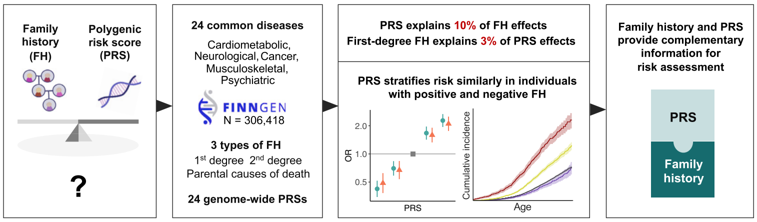

Authors

Nina Mars, Joni V. Lindbohm, Pietro della Briotta Parolo, ..., Aarno Palotie, FinnGen, Samuli Ripatti

Correspondence

samuli.ripatti@helsinki.fi

Leveraging family relationships, nationwide registries, and genome-wide genotyping, Mars et al. systematically compared two measures of inherited disease risk across 24 diseases: family history and polygenic risk scores. The measures provided complementary information for risk assessment, demonstrating opportunities for a more comprehensive way of assessing inherited risk in clinical care.

Mars et al., 2022, The American Journal of Human Genetics *109*, 2152–2162
December 1, 2022 © 2022 The Authors.
https://doi.org/10.1016/j.ajhg.2022.10.009

ARTICLE

Systematic comparison of family history and polygenic risk across 24 common diseases

Nina Mars,[1,2] Joni V. Lindbohm,[3,4,5] Pietro della Briotta Parolo,[1] Elisabeth Widén,[1] Jaakko Kaprio,[1,3] Aarno Palotie,[1,2,6] FinnGen,[7] and Samuli Ripatti[1,3,5,*]

Summary

Family history is the standard indirect measure of inherited susceptibility in clinical care, whereas polygenic risk scores (PRSs) have more recently demonstrated potential for more directly capturing genetic risk in many diseases. Few studies have systematically compared how these overlap and complement each other across common diseases. Within FinnGen (N = 306,418), we leverage family relationships, up to 50 years of nationwide registries, and genome-wide genotyping to examine the interplay of family history and genome-wide PRSs. We explore the dynamic for three types of family history across 24 common diseases: first- and second-degree family history and parental causes of death. Covering a large proportion of the burden of non-communicable diseases in adults, we show that family history and PRS are independent and not interchangeable measures, but instead provide complementary information on inherited disease susceptibility. The PRSs explained on average 10% of the effect of first-degree family history, and first-degree family history 3% of PRSs, and PRS effects were independent of both early- and late-onset family history. The PRS stratified the risk similarly in individuals with and without family history. In most diseases, including coronary artery disease, glaucoma, and type 2 diabetes, a positive family history with a high PRS was associated with a considerably elevated risk, whereas a low PRS compensated completely for the risk implied by positive family history. This study provides a catalogue of risk estimates for both family history of disease and PRSs and highlights opportunities for a more comprehensive way of assessing inherited disease risk across common diseases.

Introduction

Family history (FH) is a risk factor in most common, non-communicable diseases.[1] With multiple advantages, including low cost and non-invasiveness, it captures both genetic and non-genetic familial risk and is therefore widely applied for risk stratification and health promotion. Common clinical applications include assessment of FH of breast cancer for targeted screening, earlier initiation of cardiovascular disease prevention, and evaluating the likelihood of rheumatic disease in individuals with inflammatory arthritis.[2–4] Despite the advantages, assessment of FH also has important limitations in capturing inherited disease risk. Many individuals with common diseases have no FH, or may not know the diseases their relatives have, and the same level of familial risk is assigned to all relatives of similar degree. The accuracy of FH is fairly low owing to factors such as recall bias, and sensitivity to wording in queries may lead to misinterpretation of risk.[5,6] With average family sizes declining in many developed countries,[7] FH will also provide increasingly less information for a comprehensive assessment of familial risk.

The algorithmic developments and rapid growth in genome-wide genetic testing provide a more personalized approach for measuring genetic susceptibility through polygenic risk scores (PRSs).[8,9] PRSs employ information from large-scale genetic screens comparing allele frequencies in thousands of individuals with a disease to healthy controls and have identified numerous genetic loci for virtually all common diseases.[10] To estimate polygenic risks, the common genetic variation and the effects on the disease risks are integrated into a single metric, the PRS. The effectiveness of PRSs in risk stratification has been demonstrated for many diseases, with predictive value demonstrated alongside established clinical risk assessment tools.[11] Similarly, PRSs modify risk among individuals with high-risk variants and identify high-risk individuals for whom existing prediction tools are suboptimal.[11–16]

Given the initial expense of implementing PRS estimation in a clinical setting relative to the seemingly simple questions pertaining to family history, systematic evaluation of the independent added benefit of PRS across common diseases is essential. Studies on individual diseases have observed fairly independent effects of PRS and first-degree FH,[11,15,17–27] but few studies have systematically compared the relative contributions and overlap of PRS and FH across different types of familial risk, across varying genetic architectures, and across a wide range of diseases. Moreover, only a few studies have used genome-wide PRSs, although these contemporary PRSs containing a large number of variants have demonstrated improved

[1]Institute for Molecular Medicine Finland, FIMM, HiLIFE, University of Helsinki, Helsinki, Finland; [2]Stanley Center for Psychiatric Research, Broad Institute of MIT and Harvard, Cambridge, MA, USA; [3]Clinicum, Department of Public Health, University of Helsinki, Helsinki, Finland; [4]Department of Epidemiology and Public Health, University College London, London, UK; [5]Broad Institute of MIT and Harvard, Cambridge, MA, USA; [6]Analytic and Translational Genetics Unit, Department of Medicine, Massachusetts General Hospital, Boston, MA, USA
[7]Further details can be found in Table S10
*Correspondence: samuli.ripatti@helsinki.fi
https://doi.org/10.1016/j.ajhg.2022.10.009.
© 2022 The Authors. This is an open access article under the CC BY license (http://creativecommons.org/licenses/by/4.0/).

performance beyond PRSs with less variants due to high polygenicity in common diseases.[13,28–30] Here we study the interplay of first- and second-degree FH, parental causes of death, and genome-wide PRSs for 24 diseases using FinnGen (N = 306,418), showing that FH and PRSs are largely independent and provide complementary information in risk assessment.

Material and methods

Participants and diseases

This observational study uses FinnGen study Data Freeze 7, a collection of 306,418 adults (age ≥18) from epidemiological cohorts, disease-based cohorts, and hospital biobanks (Table S1). We used three binary definitions for FH: (1) any type of first-degree FH (FH_{1st} morbidity or mortality), (2) any type of second-degree FH (FH_{2nd}), and (3) parental cause of death (FH_P). Both for the index individual and their relatives (i.e., how FH was obtained), cases were identified through nationwide healthcare registries. The first two definitions were mapped using the genetic information to identify pairs of related FinnGen participants, whereas information on parental causes of death was available for all FinnGen participants. The 24 diseases were chosen based on availability of large published genome-wide association studies (GWASs) with full summary statistics available for genome-wide PRSs (Table S2). Disease definitions are in Table S3. Registry follow-up ended on December 31, 2019, with parental causes of death available until December 31, 2018. For FH_P, we studied 15 out of the 24 diseases, identifying causes of death (immediate, contributing, and underlying causes of death). The study was conducted in accordance with the ethical standards of the institutional and national research committees, with participants providing informed consent. Ethics statement and details on genotypes, PRS generation, and inference of relatedness are in the supplemental material and methods.

Polygenic risk scores

For each of the 24 diseases, we constructed disease-specific PRSs in a systematic manner. PRS-CS[31] was used for inferring posterior effect sizes from the GWASs listed in Table S2, with the number of cases in the GWASs ranging from 3,769 (epilepsy) to 567,460 (eGFR used for chronic kidney disease). The 1000 Genomes Project European sample (N = 503) served as the external linkage disequilibrium (LD) reference panel.[32] The posterior effect sizes were then used for calculating the PRSs.

The PRS was analyzed primarily as a continuous variable, with selected analyses applying either a (1) binary definition of FH, with high PRS defined as a PRS in the top decile of the distribution, with the rest as the reference group, or (2) PRS categories 0%–10%, 10%–20%, 20%–40%, 40%–60%, 60%–80%, 80%–90%, and 90%–100%, with the reference group being 40%–60%. To assess the impact of high versus low PRS, the reference category was 33rd to 90th percentiles, and low PRS was defined as the lowest tertile of the distribution, to allow for a sufficient number of cases with low PRSs.

Statistical analysis

Associations between FH, PRS, and risk of disease were assessed with logistic regression, with models adjusted for sex, birth year, genotyping array, cohort, and the first ten genetic principal components of ancestry. Interactions between FH and the continuous PRS (scaled to zero mean and unit variance) were assessed by introducing their interaction term to the regression model, assessing statistical significance set at a p value threshold of 0.0013 (Bonferroni correction for 24 + 15 tests). Cumulative incidences by age 80 were estimated with Kaplan-Meier survival curves (R package survminer). Statistical analyses were performed using R, version 4.1.0.

Results

FinnGen comprises 306,418 individuals (56.3% women; mean age 59.8 at the end of follow-up in 2019, SD 17.3). For the 24 diseases, FH was defined as (1) first-degree family history, FH_{1st} (morbidity or mortality), (2) second-degree family history, FH_{2nd}, and (3) parental cause of death, FH_P. Each identifies the relatives' diagnoses systematically through nationwide registries, including the hospital discharge registry (available from 1968 onward), causes of death registry (from 1964), and the Finnish Cancer Registry (from 1953). FH_{1st} and FH_{2nd} leverage the genetic relatedness within FinnGen: out of 306,418 individuals, we identified 39,444 with first-degree relative pairs based on the KING kinship coefficient[33] (see supplemental material and methods for details; 60.3% women; mean age 53.0, SD 16.5; parent-offspring relationship in 19,261 individuals, full-sibling relationship in 20,183). For breast cancer, we studied only women (15,281 individuals, mother-daughter relationship in 7,770; full sisters in 7,511), and for prostate cancer, only men (9,473 individuals; father-son relationship in 3,932; full brothers in 5,541). Similarly, we identified 47,154 individuals with a second-degree relative in the dataset (63.2% women, mean age 47.5, SD 15.0; N = 18,973 for breast cancer; N = 12,355 for prostate cancer). Parental causes of death (FH_P) were linked through the causes of death registry available from 1964 to 2019, and we excluded 78,436 whose parents had both died before 1964 or who had missing data on both parents (e.g., due to emigration), resulting in 227,982 individuals (mean age 53.6, SD 15.1; N = 133,653 for breast cancer; N = 94,329 for prostate cancer; 70,225 [30.1%] with one and 73,299 [32.2%] with two dead parents). See Figure S1 for study flow diagram.

Family history and risk of disease

First, we systematically evaluated the effects of FH on risk of disease. Figure 1 shows the prevalence of the diseases and the prevalence and effect sizes for positive FH. The most common diseases were cardiometabolic diseases, followed by knee osteoarthritis and hypothyroidism. Positive FH_{1st} was significantly associated with higher risk of disease in all diseases except stroke. The effect sizes ranged from odds ratio (OR) 3.25 (95% confidence interval, CI, 2.41–4.37) in chronic kidney disease to OR 1.17 (0.98–1.39) in stroke (Table S4). For FH_{2nd}, 18 of 24 diseases showed evidence of an association, with their effect sizes ranging from OR 1.85 (1.19–2.89) in colorectal cancer to OR 1.17 (1.09–1.25) in hypertension (Table S5). Compared to FH_{1st}, the effect sizes for FH_{2nd} were on average 69.1%

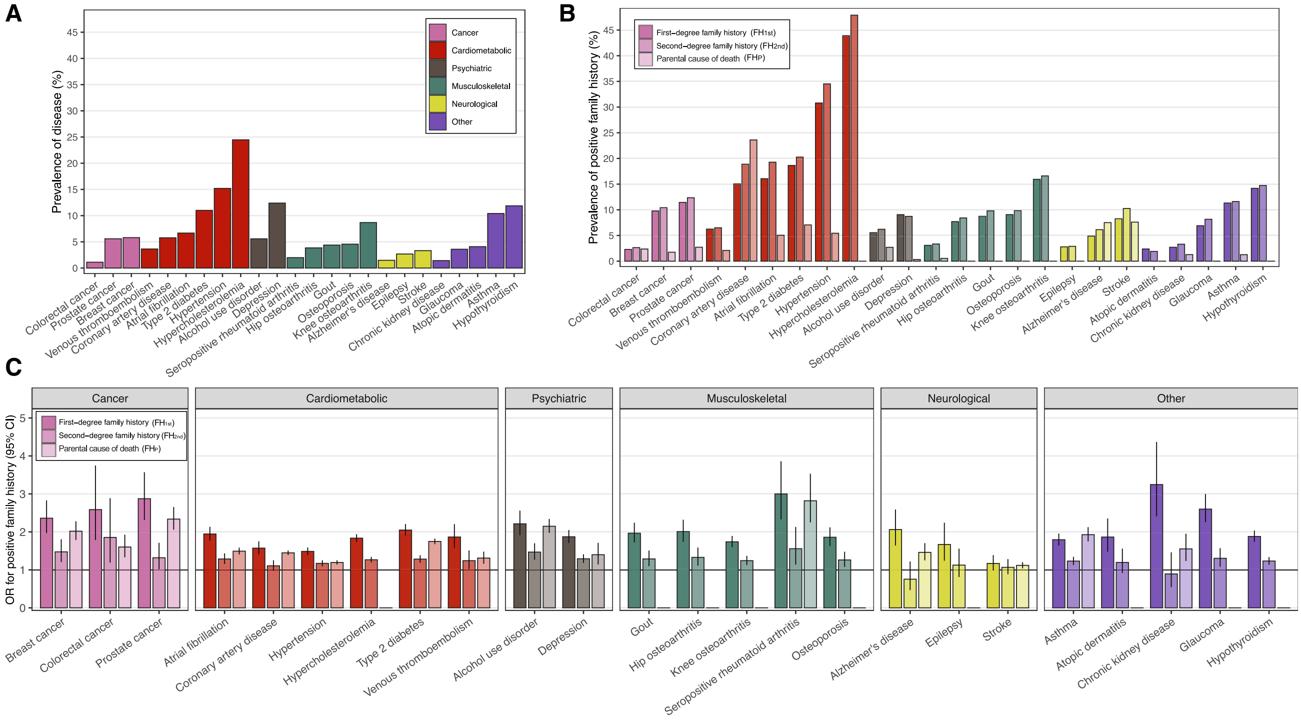

Figure 1. Disease prevalence and prevalence and effect sizes of positive family history
(A) Disease prevalence in individuals for whom we studied risk of first-degree family history.
(B) Prevalence of first-degree family history (left column), second-degree family history (middle column), and parental cause of death (right column).
(C) Effect size of first-degree family history (left column), second-degree family history (middle column), and parental cause of death (right column) with respective diseases. For parental causes of death, we studied 15 out of the 24 diseases.
Sample size in (A): total N = 39,444, N = 15,281 for breast cancer, N = 9,473 for prostate cancer. Sample sizes in (B) and (C): first-degree family history as in (A); second-degree family history total N = 47,154, N = 18,973 for breast cancer, N = 12,355 for prostate cancer; and parental causes total N = 227,982, N = 133,653 for breast cancer, N = 94,329 for prostate cancer. Odds ratios (ORs) were obtained from logistic regression models adjusted for sex (except for breast and prostate cancer), birth year, genotyping array, cohort, and the first ten genetic principal components of ancestry.

lower (SD 25.0%; calculated from log odds), i.e., a third of the effect of FH_{1st}. For FH_P, out of the 24 diseases, we studied 15 diseases that are well captured by causes of death and used information from all recorded causes of death (immediate, contributing, and underlying causes of death on the death certificate). For all 15 diseases, we observed an association between FH_P and risk of disease, with effect sizes ranging from OR 2.82 (2.25–3.53) in seropositive rheumatoid arthritis to OR 1.12 (1.04–1.20) in stroke (Table S6). Compared to FH_{1st}, the effect sizes for FH_P were on average 30.1% lower (SD 22.4%), i.e., two-thirds of the effect of FH_{1st}.

Overlap of family history and polygenic risk

Next, we compared the overlap between FH and PRSs. We constructed 24 genome-wide PRSs with uniform methodology using PRS-CS,[31] one for each disease (Table S2). We first compared the effect sizes per standard deviation (SD) increase for PRS and FH_{1st} (Figure 2, Table S4). The PRS was associated with elevated risk in all 24 diseases. The higher the PRS, the higher the proportion of positive FH (Figure S2). Effect sizes for the PRS ranged from OR 2.33 (95% CI 2.10–2.58) in prostate cancer to OR 1.12 (1.05–1.20) in epilepsy. Adjusting the PRS effect size with FH_{1st}, the change in effect size was small (mean decrease as log odds −3.0%, SD 1.3%). Adjusting the effect of FH_{1st} with PRS led to a mean decrease of −10.3% (SD 6.0%), i.e., PRS explained one-tenth of first-degree family history. No decrease in effect size was observed for PRS adjusting with FH_{2nd} (Table S5). We observed similar results for FH_P (Table S6; effect size decrease adjusting PRS effects with FH_P −0.7%, SD 0.6%; vice versa −14.5%, SD 9.2%). Proportional decreases in log odds by disease for all definitions of FH are in Figure 3. FH generally explained a much smaller fraction of the effect of PRS than vice versa. A similar pattern was observed categorizing the PRS and comparing high PRS (>90th percentile) to the rest of the distribution (Table S7 and Figure S3). A high PRS conferred on average similar effect sizes as FH_{1st}. The effect sizes particularly in common cancers and cardiometabolic diseases were higher for the PRS, whereas the effect sizes for psychiatric diseases were higher for FH_{1st}.

As early-onset FH is considered a particularly important familial risk factor, we also assessed the impact of FH_P divided into tertiles of age at death. The largest effect size was observed for FH_P with the lowest age tertile, in line

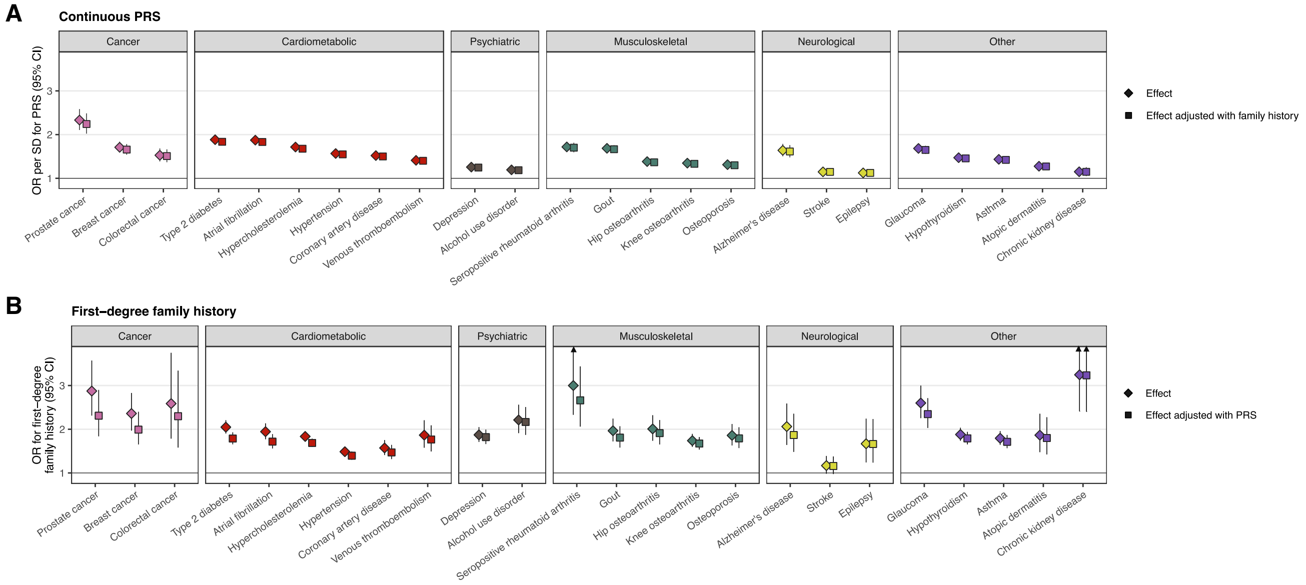

Figure 2. Cross-adjustment effects for first-degree family history (FH_{1st}), and respective polygenic risk scores (PRS)
(A and B) The impact of adjusting the PRS effect with first-degree FH_{1st} (A) and vice versa (B). The diamonds represent the unadjusted effects and the squares the adjusted effects. The PRS explained on average 10% of the effect of FH_{1st}, but FH_{1st} only 3% of the PRSs. The PRS effect is shown per one SD increase. Total N = 39,444, N = 15,281 for breast cancer, N = 9,473 for prostate cancer. Odds ratios (ORs) were obtained from logistic regression models adjusted for sex (except for breast and prostate cancer), birth year, genotyping array, cohort, and the first ten genetic principal components of ancestry.

with early-onset FH being a stronger risk factor than late-onset FH. Adjusting the PRS with this FH_P divided into age tertiles had no impact on the effect sizes of the PRSs. Adjusting this FH_P by PRS resulted in the largest effect size decreases for the youngest age tertile, but the decreases were overall small. These show that the PRS was independent of both early- and late-onset FH_P (Table S8 and Figure 4).

With formal interaction testing, we did not identify any systematic interactions between FH and PRS (Figure S4), which was further supported by observing similar PRS effect sizes in individuals with positive and negative FH_{1st} (Figure 5).

Moreover, we compared the performance of our contemporary genome-wide PRSs to previously published PRSs containing a smaller number of variants, obtained from PGS Catalog (https://www.pgscatalog.org/). Genome-wide PRSs had on average larger effect sizes (mean absolute difference in log odds 0.13 larger for genome-wide PRSs), whereby they also explained on average a larger proportion of the effect size of family history than the smaller PRSs (Figure 6).

Polygenic risk in individuals with a positive family history

Next, having assessed the overlap between FH and the PRSs, we estimated how high and low PRSs impact disease risk in individuals with positive FH_{1st}. Looking at cumulative incidence of risk of disease with the PRSs divided into three groups (high PRS >90%, average PRS 33%–90%, and low PRS <33%), we observed that a low PRS systematically compensated for the impact of positive FH_{1st}, and individuals with a combination of high PRS and positive FH_{1st} had a particularly high risk (Figure 7). Survival curves for a broader set of diseases and survival curves stratifying individuals with no FH_{1st} into similar PRS groups are in Figures S5 and S6.

Concordance of high polygenic risk in relatives

Lastly, we assessed concordance—detection of a high PRS among first- and second-degree relatives, relevant for cascade screening in relatives of individuals with high PRS. We evaluated two questions: (1) "What is the probability of having high PRS, if a relative has high PRS?" and (2) "How does this probability differ with relative's disease status?" For (1), on average 33.7% of the first-degree and 19.8% of second-degree relatives had a similarly high PRS (Figures S7 and S8). For (2), the concordance was somewhat higher with positive FH_{1st} than with negative FH_{1st}, with an average difference of 2.5% (range 0.0%–7.9%). For FH_{2nd}, no difference with disease status was observed (average 0.6%).

Discussion

Covering a large proportion of the burden of non-communicable diseases in adults, we systematically compared the overlap of polygenic risk and different types of family history, showing that they provide independent and complementary information of inherited disease susceptibility in all 24 studied diseases. PRS explained on average 10% of the effect of FH_{1st}, but FH_{1st} only 3% of the PRSs, and the

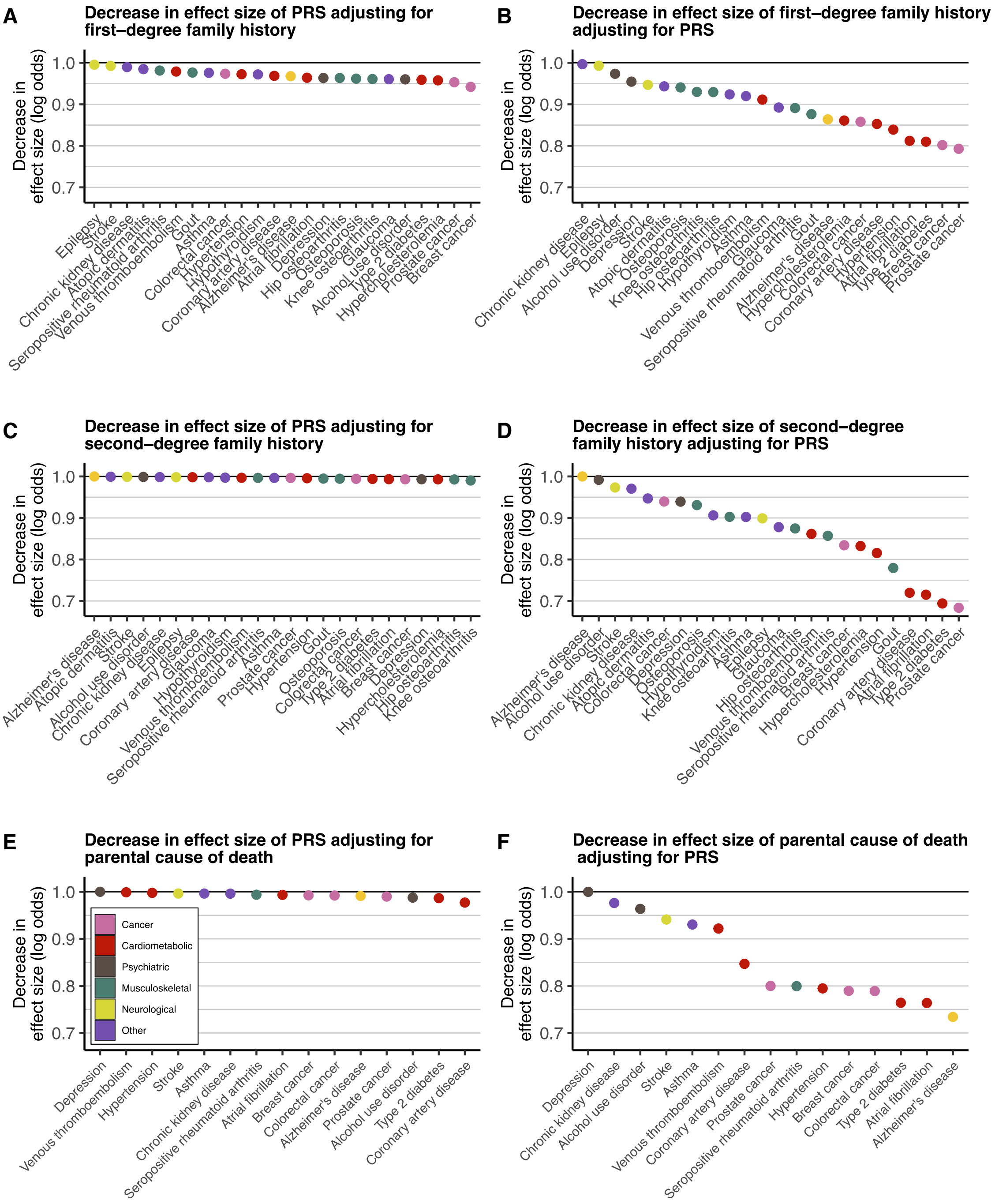

Figure 3. Cross-adjustment effect size decreases

(A–F) Proportional decreases in log odds by disease for first-degree family history, FH_{1st} (A and B), for second-degree family history, FH_{2nd} (C and D), and parental causes of death, FH_P (E and F). The left column (A, C, and E) represents decreases in effect size of high polygenic risk score (PRS, per SD) adjusting for family history. The right column (B, D, and F) represents decreases in effect size of family history adjusting for high PRS. The y axis represents the decrease in the effect size, calculated by dividing the log odds from the adjusted logistic regression model with the log odds from the non-adjusted model. For instance, in (A), the y axis represents the following quantity: (log odds of PRS adjusting for FH_{1st}) / (log odds of PRS without adjusting for FH_{1st}). In (D), the proportion of Alzheimer's disease was set at 1.00 as we did not observe any association for second-degree family history of Alzheimer's disease.

PRSs were independent of both early- and late-onset family history. The PRS estimates stratified risk similarly in individuals with and without positive FH: a high PRS conferred a considerably elevated risk, whereas a low PRS compensated for the effect of FH.

Our results are in line with previous disease-specific reports observing at most a modest attenuation in the effect of FH adjusting for PRS in cardiometabolic diseases, cancers, and depression.[11,15,17–27,34] We extend these by a systematic comparison across 24 common diseases, using

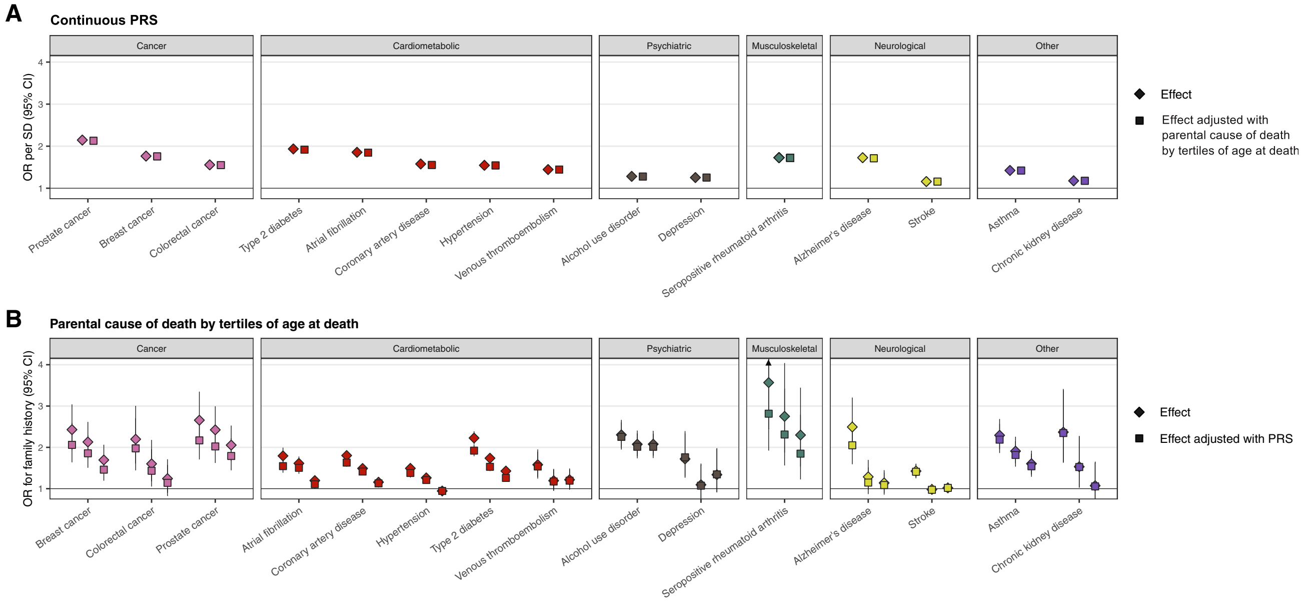

Figure 4. Polygenic risk is independent of both early- and late-onset family history

(A and B) As early-onset family history is considered a particularly important familial risk factor, we also assessed the impact of FH_P divided into tertiles of age at death. (A) Adjusting the effect of polygenic risk score (PRS; per SD) by parental causes of death (FH_P) divided into tertiles of age at death had no impact on the effect sizes of the PRSs. (B) Adjusting the effects FH_P by tertiles of age at death by PRS resulted in the largest effect size decreases for the youngest age tertile; however, for most diseases the difference by age tertile was small. The diamonds represent the unadjusted effects and the squares the adjusted effects. In (B), the effect sizes from lowest to highest age at death are displayed from left to right, and the reference group for each disease is individuals with negative FH_P. Sample size: total N = 227,982, N = 133,653 for breast cancer, N = 94,329 for prostate cancer. Odds ratios (ORs) were obtained from logistic regression models adjusted for sex (except for breast and prostate cancer), birth year, genotyping array, cohort, and the first ten genetic principal components of ancestry. Age limits for tertiles of FH_P and the number of individuals with parental cause of death in each tertile are reported in Table S8.

genome-wide PRSs generated with uniform methodology, by measuring FH uniformly through nationwide healthcare registries, and by leveraging genetic relatedness. Our results show that effects of FH and polygenic risk scores are independent, indicating that these measures complement each other for assessment of inherited disease risk. Compared to prevention guidelines that do not recommend use of PRS when FH is available,[3] these results provide important data supporting the use of PRS for improving risk assessment of several diseases with major public health importance.

The largely independent effects have several potential explanations. In addition to capturing shared DNA, FH measures non-genetic exposures and behaviors shared by families. In contrast, PRSs capture each person's unique combinations of common, disease-associated genetic variants, including genetic risk variation not shared by the relatives. PRSs can be measured in any phase of life, whereas FH relies on disease events having actualized in relatives with most utility in late-onset diseases. FH also assigns a similar risk for all relatives of the same degree, despite everyone carrying a unique set of genetic variants measurable through PRSs. Our observation of independent effects is also in line with earlier reports showing the importance of FH of breast and ovarian cancers in individuals with high-risk variants in *BRCA1* and *BRCA2*.[35]

Genetic information is typically considered in clinical care only when evidence-based prevention strategies to attenuate risk are available.[36] For instance, risk assessment of cancers has long tradition of comprehensive ascertainment of FH to identify familial clustering[37] when targeted interventions and screening tools are available.[2,38] Our results indicate that PRSs could be used to refine risk assessment of breast, prostate, and colorectal cancer, even when information about FH is available. In glaucoma, a high PRS and FH had equal and largely independent effects, but only FH is currently used for assessing risk of glaucoma in individuals with ocular hypertension.[39] The risk of coronary artery disease and type 2 diabetes can be decreased by lifestyle interventions and medications, and FH is commonly used for assessing their risk.[3,40] For both diseases, we observed larger effects for high PRS than for FH. Moreover, a high PRS may identify individuals more likely to benefit from preventive treatments: for coronary artery disease, a high PRS can result in higher relative efficacy of statins and disclosing PRS risk together with traditional risk factors can motivate lifestyle changes.[41–43] In contrast, stroke PRSs and FH show lower effect sizes than other cardiovascular diseases, likely owing to the heterogeneity of the disease and differing etiological patterns of stroke subtypes.[44,45]

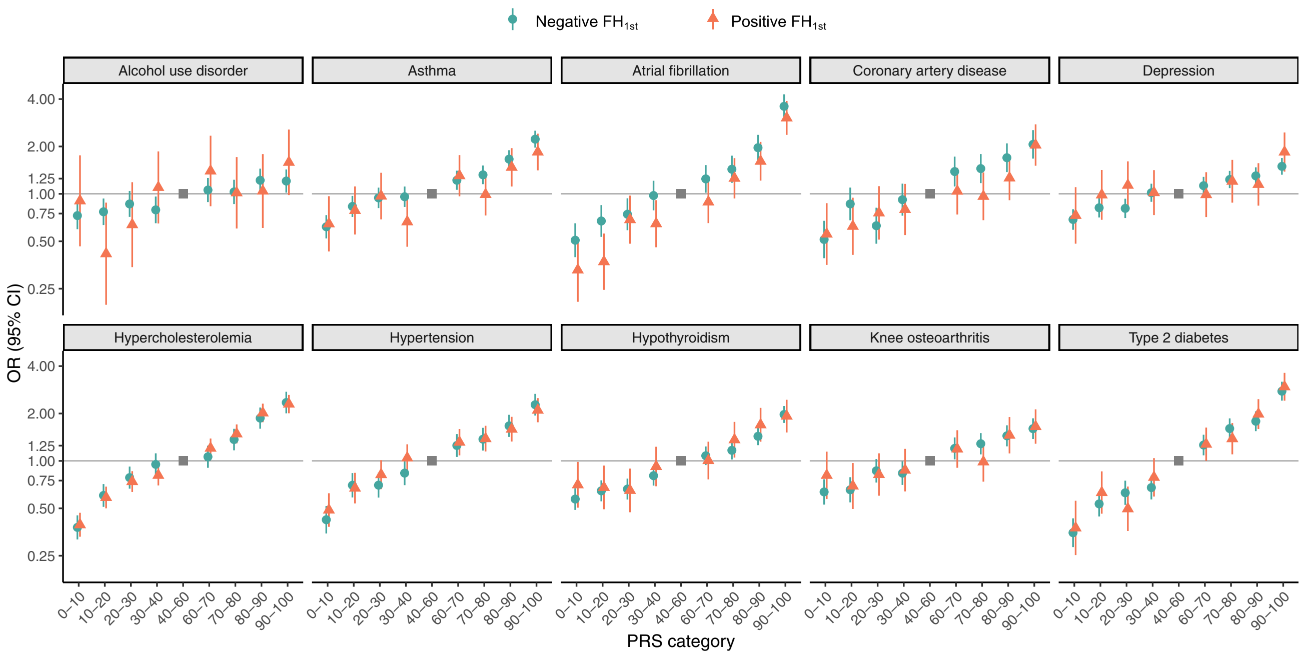

Figure 5. Effect sizes of polygenic risk scores stratified by first-degree family history (FH_{1st})
The effect sizes were calculated for the 10 most prevalent diseases. The gray box represents the reference category.

This study has multiple strengths. FH was assessed systematically and comprehensively by using linkages to high-quality nationwide registries, including hospital discharges, causes of death, and medication reimbursement registries and by overcoming several limitations of self-reported FH, such as recall bias, sensitivity to wording, and inter-individual differences in knowledge about FH.[5,6,46] We report effects of FH for disorders challenging to capture precisely from self-reported data, such as alcohol use disorder and atrial fibrillation, and show effects for diseases less studied in the field of PRSs, including glaucoma and hypothyroidism. Unlike FH, extremes of PRSs can also be used to identify individuals at particularly high or low risk. Moreover, our contemporary genome-wide PRSs had on average much larger effect sizes than previously published PRSs that are based on a smaller number of variants. This observation highlights the complex genetic architecture of common diseases and is in line with earlier reports on individual diseases.[29,30] FinnGen's wide age range is a key strength of the study, allowing systematic comparison of polygenic risk and FH across 24 diseases. Our results are also supported by quantitative genetic theory.[47,48] Average concordances of a high PRS among first- and second-degree relatives was 33.7% and 19.8%, in line with estimates on cardiometabolic diseases in UK Biobank[49] and in agreement with theoretically derived concordance estimates of 32.4% and 19.3%.[48] Moreover, the study provides catalogue of risk estimates for both FH of disease and PRSs in a large-scale biobank study.

The study was limited to individuals of European ancestry, among whom current PRSs have the highest utility.[50] Although our recording of FH_{1st} and FH_{2nd} was primarily based on only one relative, FH estimates are well in line with earlier reports from epidemiological cohorts and large registry studies (Table S9). For some diseases such as breast and prostate cancer, our effect sizes for FH were slightly larger than previously reported estimates, which may reflect the higher precision of registry-obtained family history compared to self-reported family history. As information on FH_P was available for all individuals, analyses on FH_P strengthen the results and conclusions by providing a complementary source of data that does not have the same limitations as the FH_{1st} and FH_{2nd}, which rely on inference of genetic relatedness. Not being able to account for family size may under- or overestimate the clinical impact of family history. Although the various registries are efficient in capturing disease diagnoses, milder disease forms such as mild osteoarthritis or atopic dermatitis may remain uncaptured. Similarly, common conditions such as depression or alcohol use disorder are often underreported unless severe or contributing to somatic pathologies. With over half of the study participants in the dataset ascertained from hospital biobanks or disease cohorts, the data are somewhat enriched in individuals with diseases, resulting in cumulative incidences that may not be fully generalizable to the population.

In conclusion, we studied the interplay of family history and genome-wide PRSs, systematically comparing effects across 24 common diseases. The effects of family history and PRS were largely independent, and the pattern was observed across the diseases. We demonstrate that polygenic risk and family history are not interchangeable measures of genetic susceptibility. Instead, they provide complementary information, bringing opportunities for a

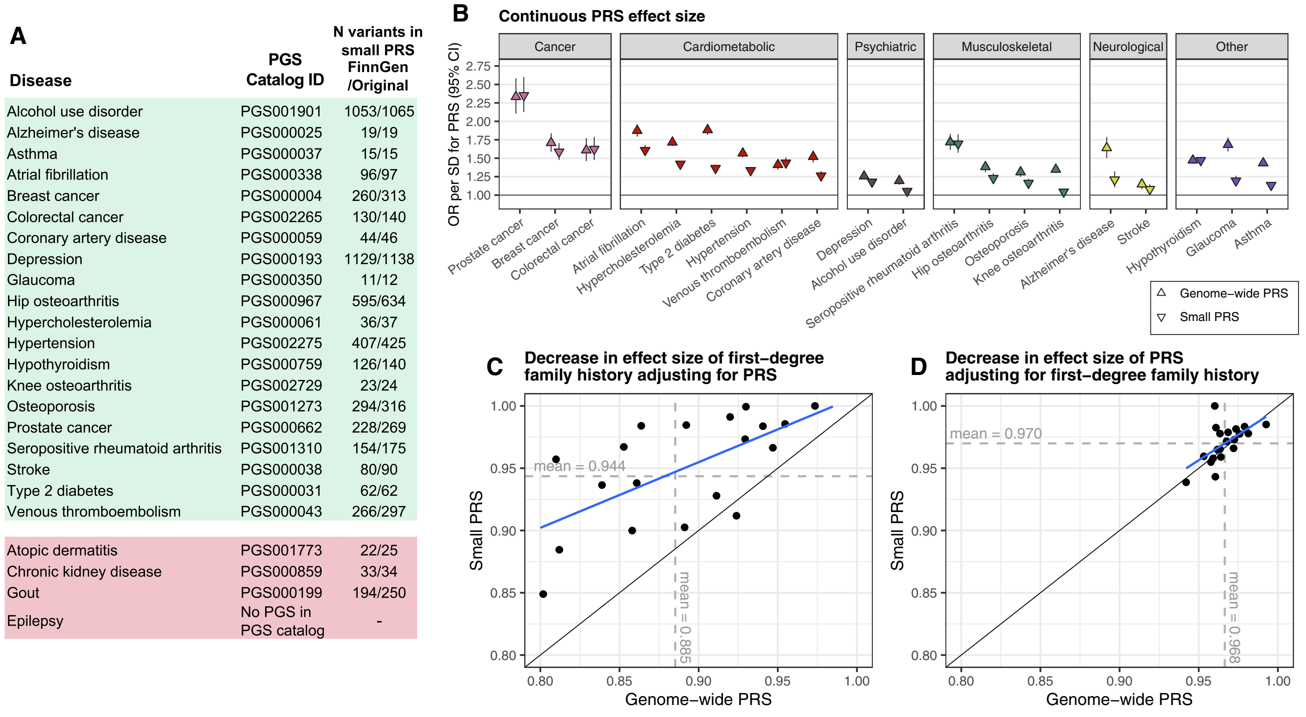

Figure 6. Comparison of our contemporary genome-wide PRSs to previously published PRSs containing a smaller number of variants
(A) Weights for the small PRSs were obtained from PGS Catalog (www.pgscatalog.org). The PRSs in green were associated with the respective endpoint in FinnGen, carried on to further comparisons. The PRSs in red showed no associations with their respective endpoints and were excluded from further analyses. No PRS for any type of epilepsy was found in PGS Catalog.
(B) Comparison of PRS effect sizes for the genome-wide PRSs and the small PRSs.
(C) Proportional decreases in effect size of first-degree family history adjusting with the PRS, showing adjustments with the genome-wide PRSs on x axis and adjustment with the small PRSs on y axis.
(D) Proportional decreases in effect size of PRS adjusting for first-degree family history, showing adjustments with the genome-wide PRSs on x axis and adjustment with the small PRSs on y axis. Similar to Figure 3, the proportional decreases in (C) and (D) represent decreases in log odds. Total N = 39,444, N = 15,281 for breast cancer, N = 9,473 for prostate cancer.

more comprehensive way of assessing inherited risk. A PRS can be calculated early in life to serve as risk indicator in individuals without family history of disease, while also providing effective risk stratification among individuals with positive family history.

Data and code availability

The FinnGen data may be accessed through Finnish Biobanks' FinBB portal (www.finbb.fi; email: info.fingenious@finbb.fi). Download links for the GWAS summary statistics used for constructing PRSs are provided in Table S2. The weights for our polygenic risk scores are available at PGS Catalog (https://www.pgscatalog.org/, publication ID PGP000364) with the PGS Catalog IDs listed in Table S2.

Supplemental information

Supplemental information can be found online at https://doi.org/10.1016/j.ajhg.2022.10.009.

Acknowledgments

We thank the researchers who have openly shared the GWAS summary statistics that were used in this study to generate PRSs. Full FinnGen acknowledgments and FinnGen funders are provided in the supplemental acknowledgments. This work has been supported by Academy of Finland (grant number 331671 to N.M., 285380 to S.R., 128650 to A.P., 308248 to J.K.), Academy of Finland Center of Excellence in Complex Disease Genetics (grant number 312062 to S.R., 312074 to A.P., 312073 to J.K.), European Union's Horizon 2020 research and innovation program under grant agreement No 101016775, the Sigrid Jusélius Foundation (to S.R. and A.P.), University of Helsinki HiLIFE Fellow grants 2017-2020 (to S.R.), the Finnish Innovation Fund Tekes (grant number 2273/31/2017 to E.W.), Foundation and the Horizon 2020 Research and Innovation Program (grant number 667301 (COSYN) to A.P.). J.V.L. was supported by Academy of Finland (grant number 311492), Helsinki Institute of Life Science (H970), and Päivikki and Sakari Sohlberg Foundation.

Author contributions

N.M., J.V.L, and S.R. conceived and designed the study. N.M. and P.d.B.P. carried out the statistical and computational analyses with advice from S.R. and J.V.L. Quality control of the data was carried out by N.M. and P.d.B.P. All authors provided critical input to interpretation of the data. The manuscript was written and revised by N.M. and S.R., with comments from all of the co-authors. All co-authors have approved the final version of the manuscript.

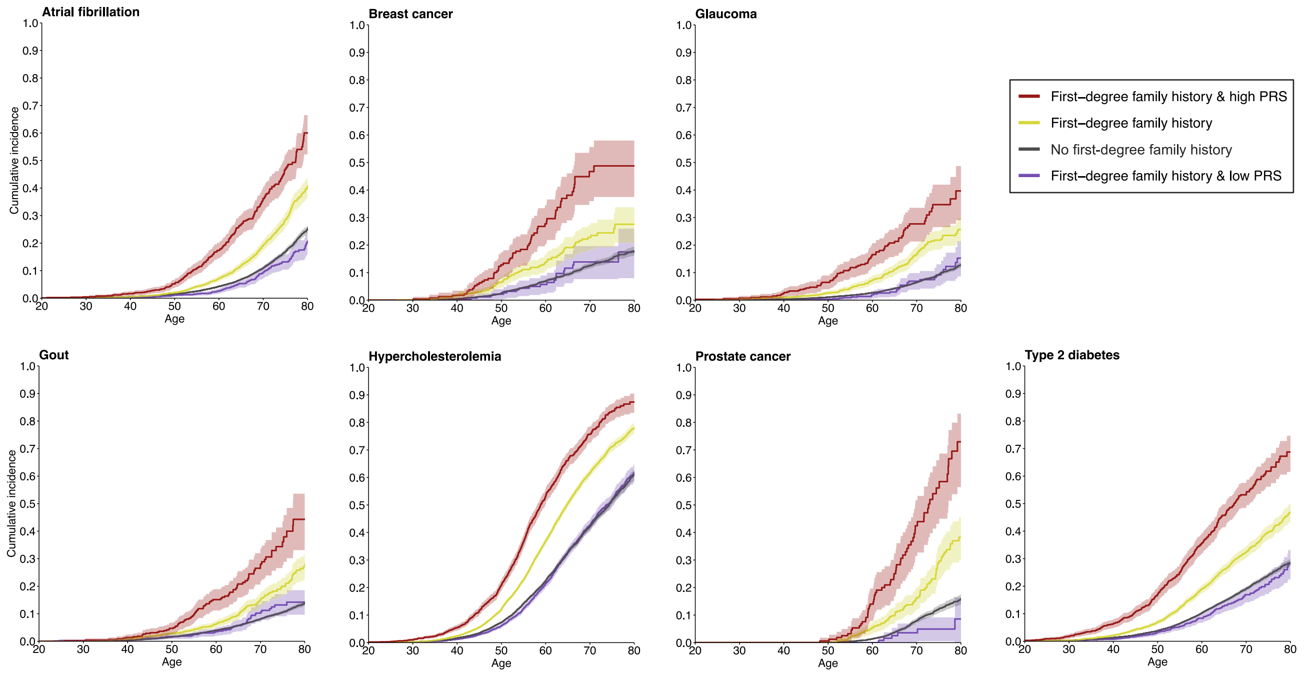

Figure 7. The impact of polygenic risk on disease risk in individuals with positive family history
The survival curves show cumulative incidences for individuals with positive first-degree family (FH_{1st}), stratified by level of polygenic risk score (PRS). High PRS was defined as top decile of the PRS distribution and low PRS as the bottom tertile of the PRS distribution. The figure shows results for the five diseases with the largest effect sizes for PRS, and for breast and prostate cancer. Survival curves for a broader set of diseases, and survival curves stratifying individuals with no FH_{1st} into similar PRS groups are in Figures S5 and S6. Total N = 39,444, N = 15,281 for breast cancer, N = 9,473 for prostate cancer. Analyses were performed for diseases with an OR >2 for high PRS in Table S7 and over 10 cases in each subgroup, excluding Alzheimer's disease due to its average onset late in life.

Declaration of interests

A.P. is a member of the Pfizer Genetics Scientific Advisory Panel.

Received: July 6, 2022
Accepted: October 17, 2022
Published: November 7, 2022

References

1. Valdez, R., Yoon, P.W., Qureshi, N., Green, R.F., and Khoury, M.J. (2010). Family history in public health practice: a genomic tool for disease prevention and health promotion. Annu. Rev. Public Health *31*, 69–87. 61 p following 87.
2. Daly, M.B., Pal, T., Berry, M.P., Buys, S.S., Dickson, P., Domchek, S.M., Elkhanany, A., and Friedman, S. NCCN Clinical Practice Guidelines in Oncology: Genetics/Familial High-Risk Assessment: Breast, Ovarian, and Pancreatic Version 2.2021. NCCN.org.
3. Piepoli, M.F., Hoes, A.W., Agewall, S., Albus, C., Brotons, C., Catapano, A.L., Cooney, M.T., Corrà, U., Cosyns, B., Deaton, C., et al. (2016). 2016 European guidelines on cardiovascular disease prevention in clinical practice: the sixth joint task force of the european society of cardiology and other societies on cardiovascular disease prevention in clinical practice (constituted by representatives of 10 societies and by invited experts)developed with the special contribution of the European Association for cardiovascular prevention & rehabilitation (EACPR). Eur. Heart J. *37*, 2315–2381.
4. Frisell, T., Saevarsdottir, S., and Askling, J. (2016). Family history of rheumatoid arthritis: an old concept with new developments. Nat. Rev. Rheumatol. *12*, 335–343.
5. Conway-Pearson, L.S., Christensen, K.D., Savage, S.K., Huntington, N.L., Weitzman, E.R., Ziniel, S.I., Bacon, P., Cacioppo, C.N., Green, R.C., and Holm, I.A. (2016). Family health history reporting is sensitive to small changes in wording. Genet. Med. *18*, 1308–1311.
6. Wilson, B.J., Qureshi, N., Santaguida, P., Little, J., Carroll, J.C., Allanson, J., and Raina, P. (2009). Systematic review: family history in risk assessment for common diseases. Ann. Intern. Med. *151*, 878–885.
7. United Nations, Department of Economic, Affairs, Social, and Division, Population (2019). Patterns and Trends in Household Size and Composition: Evidence from a United Nations Dataset (ST/ESA/SER.A/433).
8. Lewis, C.M., and Vassos, E. (2020). Polygenic risk scores: from research tools to clinical instruments. Genome Med. *12*, 44.
9. Chatterjee, N., Shi, J., and García-Closas, M. (2016). Developing and evaluating polygenic risk prediction models for stratified disease prevention. Nat. Rev. Genet. *17*, 392–406.
10. Loos, R.J.F. (2020). 15 years of genome-wide association studies and no signs of slowing down. Nat. Commun. *11*, 5900.
11. Mars, N., Koskela, J.T., Ripatti, P., Kiiskinen, T.T.J., Havulinna, A.S., Lindbohm, J.V., Ahola-Olli, A., Kurki, M., Karjalainen, J., Palta, P., et al. (2020). Polygenic and clinical risk scores and their impact on age at onset and prediction of cardiometabolic diseases and common cancers. Nat. Med. *26*, 549–557.

12. Fahed, A.C., Wang, M., Homburger, J.R., Patel, A.P., Bick, A.G., Neben, C.L., Lai, C., Brockman, D., Philippakis, A., Ellinor, P.T., et al. (2020). Polygenic background modifies penetrance of monogenic variants for tier 1 genomic conditions. Nat. Commun. *11*, 3635.
13. Mars, N., Widén, E., Kerminen, S., Meretoja, T., Pirinen, M., della Briotta Parolo, P., Palta, P., FinnGen, Palotie, A., Kaprio, J., et al. (2020). The role of polygenic risk and susceptibility genes in breast cancer over the course of life. Nat. Commun. *11*, 6383.
14. Isgut, M., Sun, J., Quyyumi, A.A., and Gibson, G. (2021). Highly elevated polygenic risk scores are better predictors of myocardial infarction risk early in life than later. Genome Med. *13*, 13.
15. Archambault, A.N., Su, Y.R., Jeon, J., Thomas, M., Lin, Y., Conti, D.V., Win, A.K., Sakoda, L.C., Lansdorp-Vogelaar, I., Peterse, E.F.P., et al. (2020). Cumulative burden of colorectal cancer-associated genetic variants is more strongly associated with early-onset vs late-onset cancer. Gastroenterology *158*, 1274–1286.e12. e1212.
16. Conti, D.V., Darst, B.F., Moss, L.C., Saunders, E.J., Sheng, X., Chou, A., Schumacher, F.R., Olama, A.A.A., Benlloch, S., Dadaev, T., et al. (2021). Trans-ancestry genome-wide association meta-analysis of prostate cancer identifies new susceptibility loci and informs genetic risk prediction. Nat. Genet. *53*, 65–75.
17. Inouye, M., Abraham, G., Nelson, C.P., Wood, A.M., Sweeting, M.J., Dudbridge, F., Lai, F.Y., Kaptoge, S., Brozynska, M., Wang, T., et al. (2018). Genomic risk prediction of coronary artery disease in 480, 000 adults: implications for primary prevention. J. Am. Coll. Cardiol. *72*, 1883–1893.
18. Tikkanen, E., Havulinna, A.S., Palotie, A., Salomaa, V., and Ripatti, S. (2013). Genetic risk prediction and a 2-stage risk screening strategy for coronary heart disease. Arterioscler. Thromb. Vasc. Biol. *33*, 2261–2266.
19. Hughes, E., Tshiaba, P., Gallagher, S., Wagner, S., Judkins, T., Roa, B., Rosenthal, E., Domchek, S., Garber, J., Lancaster, J., et al. (2020). Development and validation of a clinical polygenic risk score to predict breast cancer risk. JCO Precis. Oncol. *4*.
20. Agerbo, E., Trabjerg, B.B., Børglum, A.D., Schork, A.J., Vilhjálmsson, B.J., Pedersen, C.B., Hakulinen, C., Albiñana, C., Hougaard, D.M., Grove, J., et al. (2021). Risk of early-onset depression associated with polygenic liability, parental psychiatric history, and socioeconomic status. JAMA Psychiatr. *78*, 387–397.
21. InterAct Consortium, Scott, R.A., Langenberg, C., Sharp, S.J., Franks, P.W., Rolandsson, O., Drogan, D., van der Schouw, Y.T., Ekelund, U., Kerrison, N.D., et al. (2013). The link between family history and risk of type 2 diabetes is not explained by anthropometric, lifestyle or genetic risk factors: the EPIC-InterAct study. Diabetologia *56*, 60–69.
22. Tada, H., Melander, O., Louie, J.Z., Catanese, J.J., Rowland, C.M., Devlin, J.J., Kathiresan, S., and Shiffman, D. (2016). Risk prediction by genetic risk scores for coronary heart disease is independent of self-reported family history. Eur. Heart J. *37*, 561–567.
23. Muranen, T.A., Mavaddat, N., Khan, S., Fagerholm, R., Pelttari, L., Lee, A., Aittomäki, K., Blomqvist, C., Easton, D.F., and Nevanlinna, H. (2016). Polygenic risk score is associated with increased disease risk in 52 Finnish breast cancer families. Breast Cancer Res. Treat. *158*, 463–469.
24. Agerbo, E., Sullivan, P.F., Vilhjálmsson, B.J., Pedersen, C.B., Mors, O., Børglum, A.D., Hougaard, D.M., Hollegaard, M.V., Meier, S., Mattheisen, M., et al. (2015). Polygenic risk score, parental socioeconomic status, family history of psychiatric disorders, and the risk for schizophrenia: a danish population-based study and meta-analysis. JAMA Psychiatr. *72*, 635–641.
25. Mavaddat, N., Michailidou, K., Dennis, J., Lush, M., Fachal, L., Lee, A., Tyrer, J.P., Chen, T.H., Wang, Q., Bolla, M.K., et al. (2019). Polygenic risk scores for prediction of breast cancer and breast cancer subtypes. Am. J. Hum. Genet. *104*, 21–34.
26. Li, H., Feng, B., Miron, A., Chen, X., Beesley, J., Bimeh, E., Barrowdale, D., John, E.M., Daly, M.B., Andrulis, I.L., et al. (2017). Breast cancer risk prediction using a polygenic risk score in the familial setting: a prospective study from the Breast Cancer Family Registry and kConFab. Genet. Med. *19*, 30–35.
27. Moll, M., Lutz, S.M., Ghosh, A.J., Sakornsakolpat, P., Hersh, C.P., Beaty, T.H., Dudbridge, F., Tobin, M.D., Mittleman, M.A., Silverman, E.K., et al. (2020). Relative contributions of family history and a polygenic risk score on COPD and related outcomes: COPDGene and ECLIPSE studies. BMJ Open Respir. Res. *7*, e000755.
28. Khera, A.V., Chaffin, M., Aragam, K.G., Haas, M.E., Roselli, C., Choi, S.H., Natarajan, P., Lander, E.S., Lubitz, S.A., Ellinor, P.T., and Kathiresan, S. (2018). Genome-wide polygenic scores for common diseases identify individuals with risk equivalent to monogenic mutations. Nat. Genet. *50*, 1219–1224.
29. Dikilitas, O., Schaid, D.J., Kosel, M.L., Carroll, R.J., Chute, C.G., Denny, J.A., Fedotov, A., Feng, Q., Hakonarson, H., Jarvik, G.P., et al. (2020). Predictive utility of polygenic risk scores for coronary heart disease in three major racial and ethnic groups. Am. J. Hum. Genet. *106*, 707–716.
30. Mars, N., Kerminen, S., Feng, Y.-C.A., Kanai, M., Läll, K., Thomas, L.F., Skogholt, A.H., della Briotta Parolo, P., Neale, B.M., Smoller, J.W., et al. (2022). Genome-wide risk prediction of common diseases across ancestries in one million people. Cell Genom. *2*, 100118.
31. Ge, T., Chen, C.Y., Ni, Y., Feng, Y.C.A., and Smoller, J.W. (2019). Polygenic prediction via Bayesian regression and continuous shrinkage priors. Nat. Commun. *10*, 1776.
32. 1000 Genomes Project Consortium, Auton, A., Brooks, L.D., Durbin, R.M., Garrison, E.P., Kang, H.M., Korbel, J.O., Marchini, J.L., McCarthy, S., McVean, G.A., and Abecasis, G.R. (2015). A global reference for human genetic variation. Nature *526*, 68–74.
33. Manichaikul, A., Mychaleckyj, J.C., Rich, S.S., Daly, K., Sale, M., and Chen, W.M. (2010). Robust relationship inference in genome-wide association studies. Bioinformatics *26*, 2867–2873.
34. Schnitzer, F., Forer, L., Schönherr, S., Gieger, C., Grallert, H., Kronenberg, F., Peters, A., and Lamina, C. (2022). Association between a polygenic and family risk score on the prevalence and incidence of myocardial infarction in the KORA-F3 study. Atherosclerosis *352*, 10–17.
35. Kuchenbaecker, K.B., Hopper, J.L., Barnes, D.R., Phillips, K.A., Mooij, T.M., Roos-Blom, M.J., Jervis, S., van Leeuwen, F.E., Milne, R.L., Andrieu, N., et al. (2017). Risks of breast, ovarian, and contralateral breast cancer for BRCA1 and BRCA2 mutation carriers. JAMA *317*, 2402–2416.
36. ACMG Board of Directors (2015). Clinical utility of genetic and genomic services: a position statement of the American

College of Medical Genetics and Genomics. Genet. Med. *17*, 505–507.

37. Bennett, R.L. (2010). The Practical Guide to Genetic Family History, 2nd edition (Wiley-Blackwell).
38. Provenzale, D., Gupta, S., Ahnen, D.J., Bray, T., Cannon, J.A., Cooper, G., David, D.S., Early, D.S., Erwin, D., Ford, J.M., et al. (2019). NCCN clinical practice guidelines in oncology: genetic/familial high-risk assessment: colorectal version 3.2019. J. Natl. Compr. Canc. Netw. *14*, 1010–1030.
39. National Institute for Health and Care Excellence (2017), Glaucoma: diagnosis and management [NICE guideline No. 81]. https://www.nice.org.uk/guidance/ng81.
40. American Diabetes Association (2021). 3. prevention or delay of type 2 diabetes: standards of medical care in diabetes-2021. Diabetes Care *44*, S34–S39.
41. Natarajan, P., Young, R., Stitziel, N.O., Padmanabhan, S., Baber, U., Mehran, R., Sartori, S., Fuster, V., Reilly, D.F., Butterworth, A., et al. (2017). Polygenic risk score identifies subgroup with higher burden of atherosclerosis and greater relative benefit from statin therapy in the primary prevention setting. Circulation *135*, 2091–2101.
42. Mega, J.L., Stitziel, N.O., Smith, J.G., Chasman, D.I., Caulfield, M., Devlin, J.J., Nordio, F., Hyde, C., Cannon, C.P., Sacks, F., et al. (2015). Genetic risk, coronary heart disease events, and the clinical benefit of statin therapy: an analysis of primary and secondary prevention trials. Lancet *385*, 2264–2271.
43. Widén, E., Junna, N., Ruotsalainen, S., Surakka, I., Mars, N., Ripatti, P., Partanen, J.J., Aro, J., Mustonen, P., Tuomi, T., et al. (2022). How communicating polygenic and clinical risk for atherosclerotic cardiovascular disease impacts health behavior: an observational follow-up study. Circ. Genom. Precis. Med. *15*, e003459.
44. Abraham, G., Malik, R., Yonova-Doing, E., Salim, A., Wang, T., Danesh, J., Butterworth, A.S., Howson, J.M.M., Inouye, M., and Dichgans, M. (2019). Genomic risk score offers predictive performance comparable to clinical risk factors for ischaemic stroke. Nat. Commun. *10*, 5819.
45. Malik, R., Chauhan, G., Traylor, M., Sargurupremraj, M., Okada, Y., Mishra, A., Rutten-Jacobs, L., Giese, A.K., van der Laan, S.W., Gretarsdottir, S., et al. (2018). Multiancestry genome-wide association study of 520, 000 subjects identifies 32 loci associated with stroke and stroke subtypes. Nat. Genet. *50*, 524–537.
46. Drews, C.D., and Greeland, S. (1990). The impact of differential recall on the results of case-control studies. Int. J. Epidemiol. *19*, 1107–1112.
47. Do, C.B., Hinds, D.A., Francke, U., and Eriksson, N. (2012). Comparison of family history and SNPs for predicting risk of complex disease. PLoS Genet. *8*, e1002973.
48. Carmi, S. (2021). Cascade screening following a polygenic risk score test: what is the disease risk of a sibling conditional on a high score in a proband?. Preprint at bioRxiv.
49. Reid, N.J., Brockman, D.G., Elisabeth Leonard, C., Pelletier, R., and Khera, A.V. (2021). Concordance of a high polygenic score among relatives: implications for genetic counseling and cascade screening. Circ. Genom. Precis. Med. *14*, e003262.
50. Martin, A.R., Kanai, M., Kamatani, Y., Okada, Y., Neale, B.M., and Daly, M.J. (2019). Clinical use of current polygenic risk scores may exacerbate health disparities. Nat. Genet. *51*, 584–591.

The American Journal of Human Genetics, Volume *109*

Supplemental information

Systematic comparison of family history

and polygenic risk across 24 common diseases

Nina Mars, Joni V. Lindbohm, Pietro della Briotta Parolo, Elisabeth Widén, Jaakko Kaprio, Aarno Palotie, FinnGen, and Samuli Ripatti

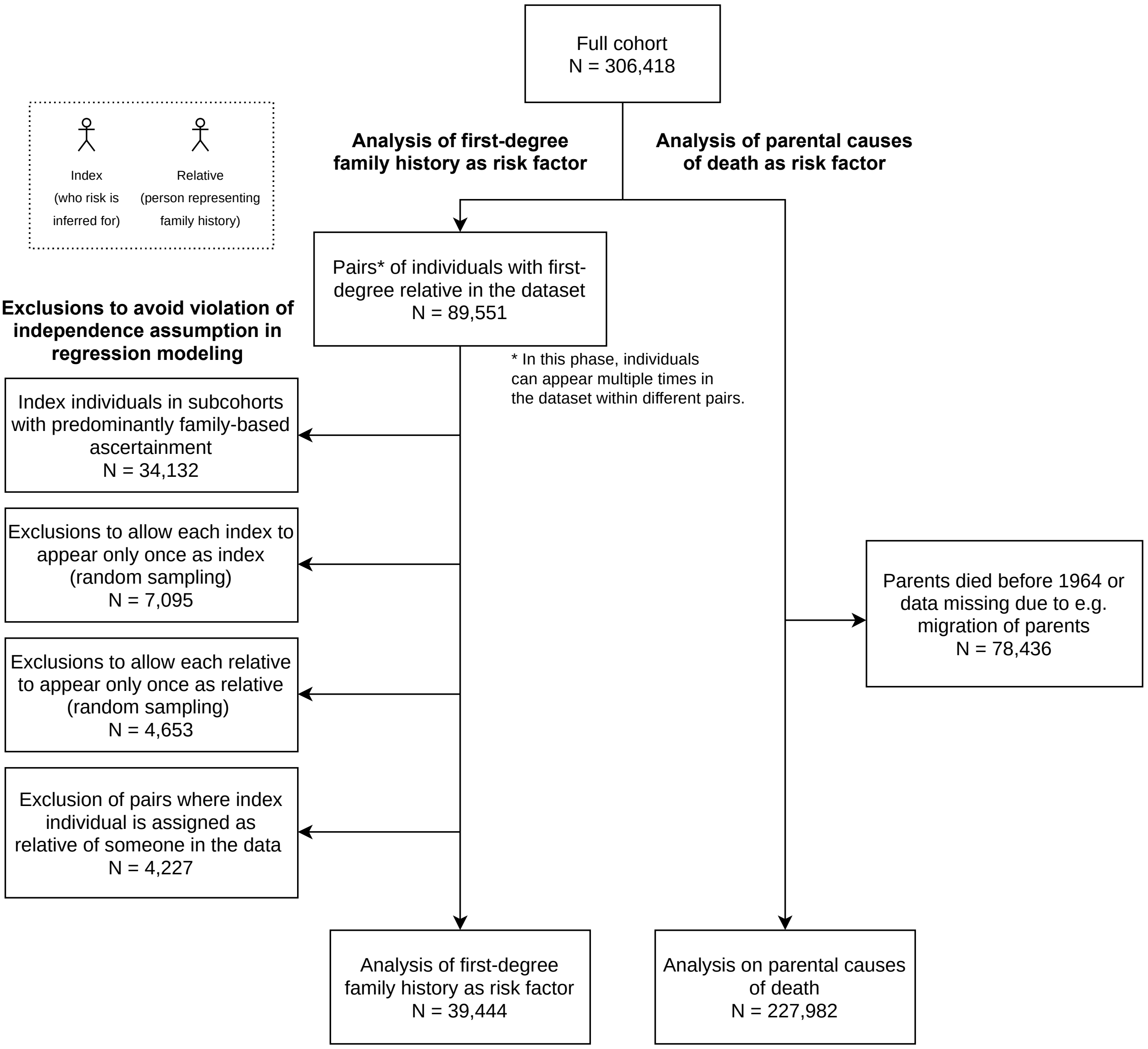

Figure S1. Study flowchart. The flowchart describes generation of datasets used for the definitions first-degree family history and parental cause of death. Having inferred kinship, the initial data structure allows individuals to appear multiple times in the dataset within different pairs. The main processing steps after this involved random exclusions, to individuals appearing multiple times on either side of the regression equation, which would violate the assumption of independence of observations. Similar steps were performed for second-degree family history as for first degree family history, starting from 118,992 pairs of individuals with a second-degree relative in the dataset and resulting in 47,154 individuals for analysis of second-degree family history as a risk factor. For breast cancer, we studied only pairs of women (15,281 individuals, parent-offspring relationship in 7,770; full-sibling relationship in 7,511). For prostate cancer, we studied only pairs of men (9,473 individuals, parent-offspring relationship in 3,932; full-sibling relationship in 5,541).

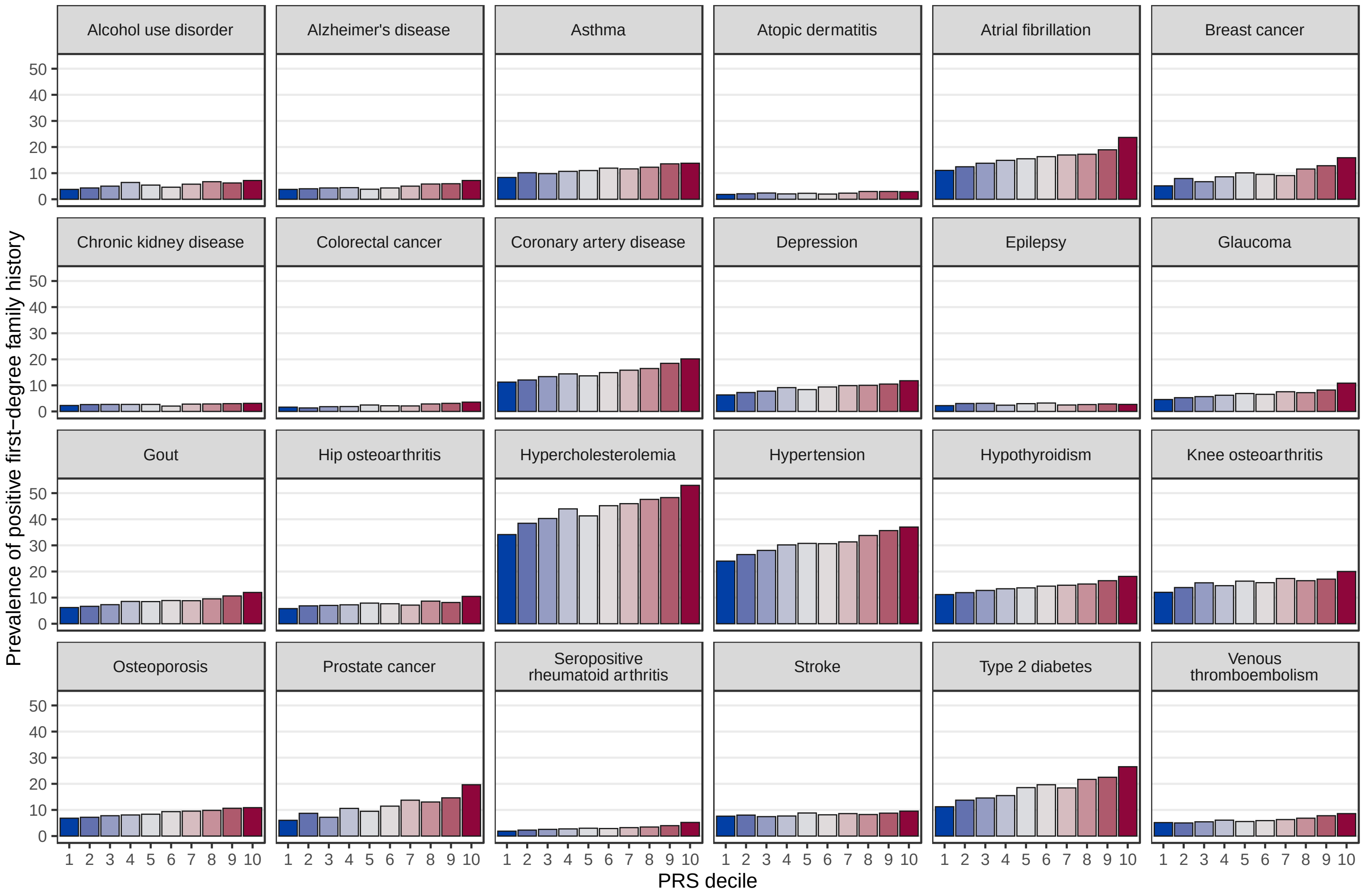

Figure S2. Prevalence of first-degree family history (FH_{1st}) by deciles of polygenic risk score (PRS). Total N = 39,444, N = 15,281 for breast cancer, N = 9,473 for prostate cancer.

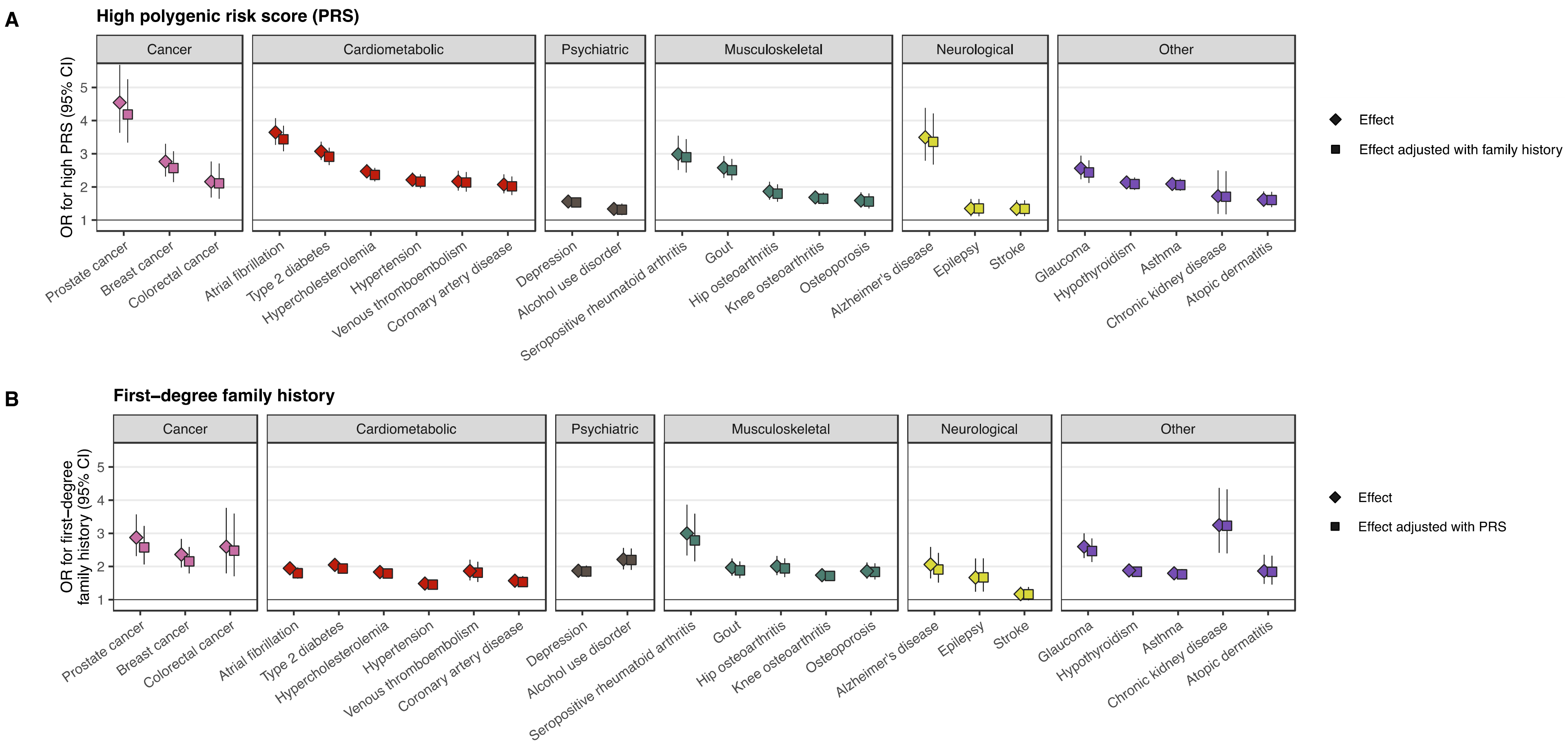

Figure S3. Cross-adjustment effects for family history and polygenic risk score (PRS) with PRS categorized. The impact of adjusting the PRS effect with first-degree family history **(**$FH_{1st,}$ **panel A)** and vice versa **(panel B)**. The diamonds represent the unadjusted effects and the squares the adjusted effects. The PRS effect size compares individuals in the top decile of the PRS distribution to the rest. Total N = 39,444, N = 15,281 for breast cancer, N = 9,473 for prostate cancer. Odds ratios (OR) were obtained from logistic regression models adjusted for sex (except for breast and prostate cancer), birth year, genotyping array, cohort, and the first ten genetic principal components of ancestry.

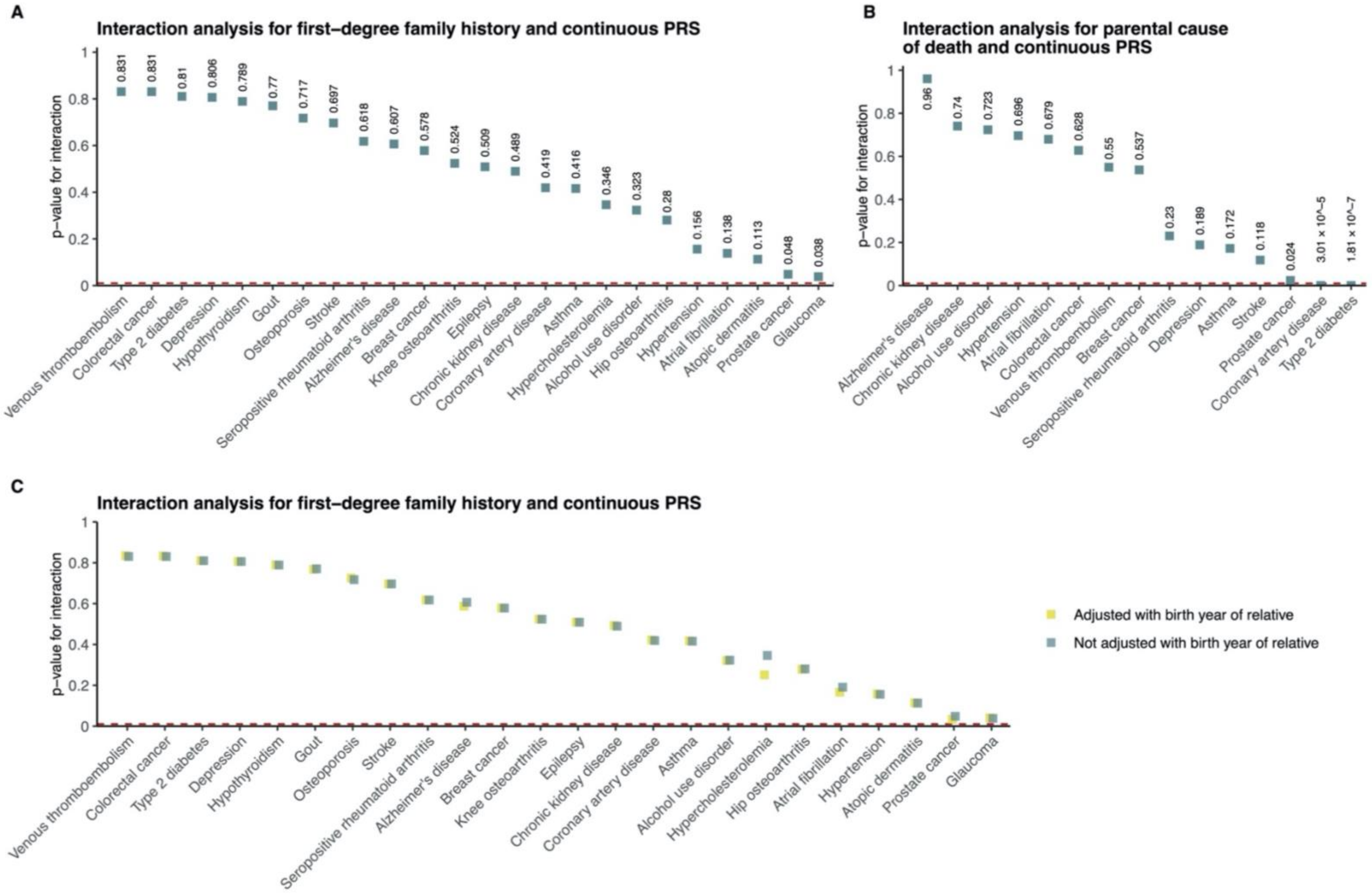

Figure S4. Interaction analysis. Interaction analysis between first-degree family history (FH_{1st}, **panel A**) or parental causes of death (FH_P, **panel B**) and respective polygenic risk scores (PRS), displaying the p-value for the interaction term on the y-axis. **Panel C** shows the results of **panel B** adjusting also for birth year of the relative. The PRSs were scaled to zero mean and unit variance and handled as continuous variables in the interaction analysis. Statistical significance set at a p-value threshold of 0.0013 (Bonferroni-correction for 24+15 tests) represented by the red line. We did not observe systematic evidence of interactions. Total N = 39,444, N = 15,281 for breast cancer, N = 9,473 for prostate cancer. The logistic regression models were adjusted for sex (except for breast and prostate cancer), birth year, genotyping array, cohort, and the first ten genetic principal components of ancestry.

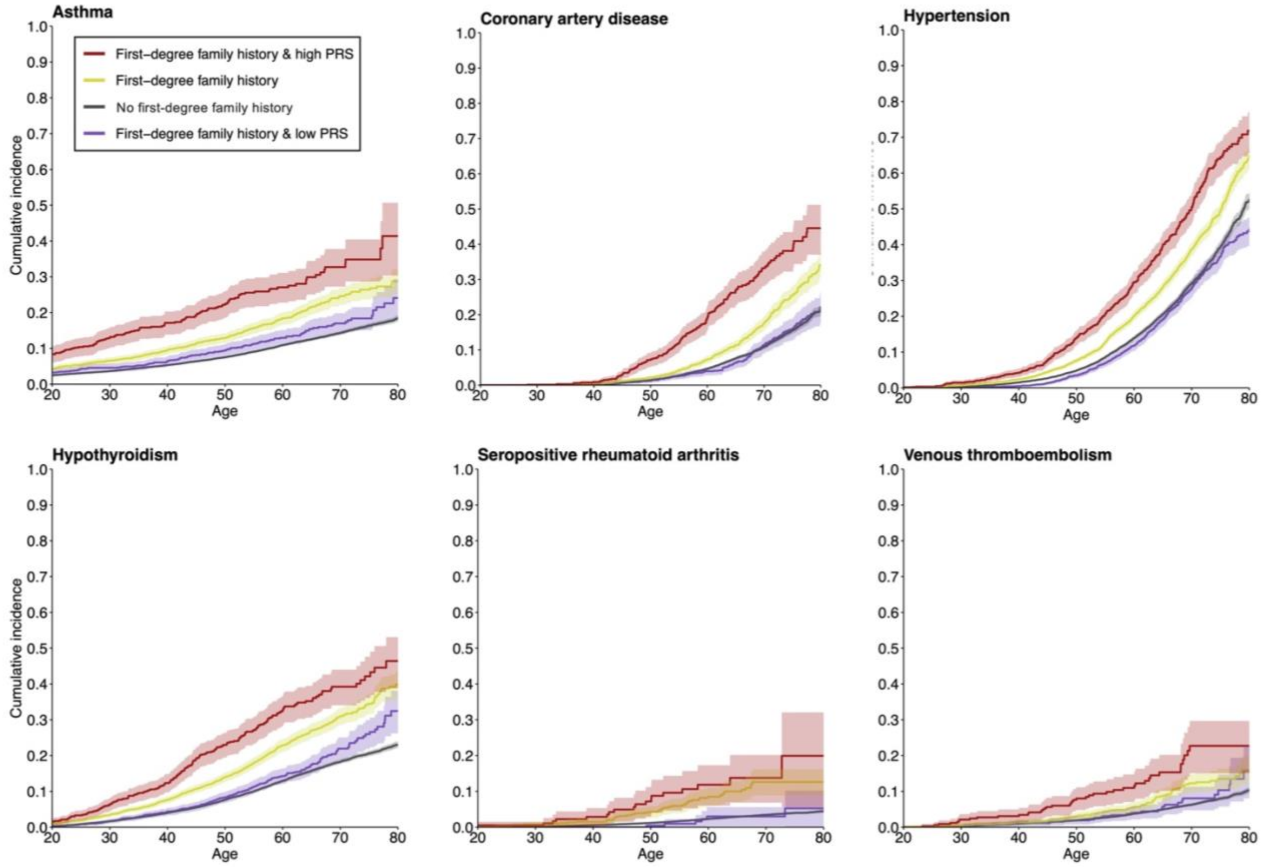

Figure S5. The impact of polygenic risk on disease risk in individuals with positive family history. Figure 6 showed results for the five diseases with the largest effect sizes for PRS, and for breast and prostate cancer, with the rest of the diseases fulfilling the criteria of an OR >2 for high PRS (Table S7) and over 10 cases in each subgroup shown here. The survival curves show cumulative incidences for individuals with positive first-degree family (FH_{1st}), stratified by level of polygenic risk score (PRS). High PRS was defined as top decile of the PRS distribution and low PRS as the bottom tertile of the PRS distribution. Total N = 39,444, N = 15,281 for breast cancer, N = 9,473 for prostate cancer.

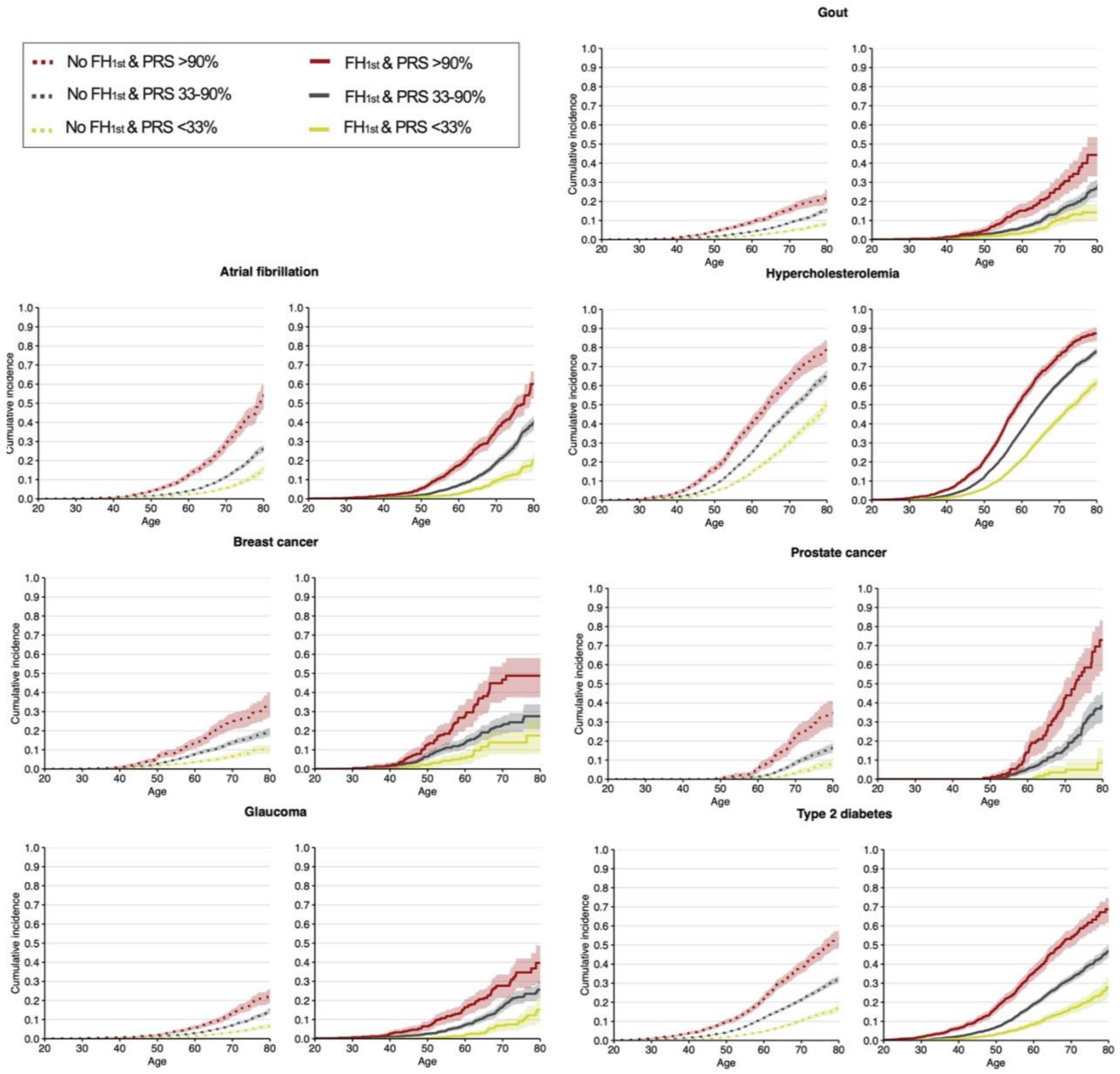

Figure S6. Polygenic risk scores (PRS) stratified by negative and positive family history. Impact of the level of PRS on cumulative incidence of disease in individuals with negative (dashed lines) and positive (solid line) first-degree family (FH_{1st}). High PRS was defined as top decile of the PRS distribution and low PRS as the bottom tertile of the PRS distribution. A low PRS compensated for the impact of positive FH_{1st}, whereas individuals with a combination of high PRS and positive FH_{1st} had a particularly high risk. The figure shows results for the five diseases with the largest effect sizes for PRS, and for breast and prostate cancer. Total N = 39,444, N = 15,281 for breast cancer, N = 9,473 for prostate cancer.

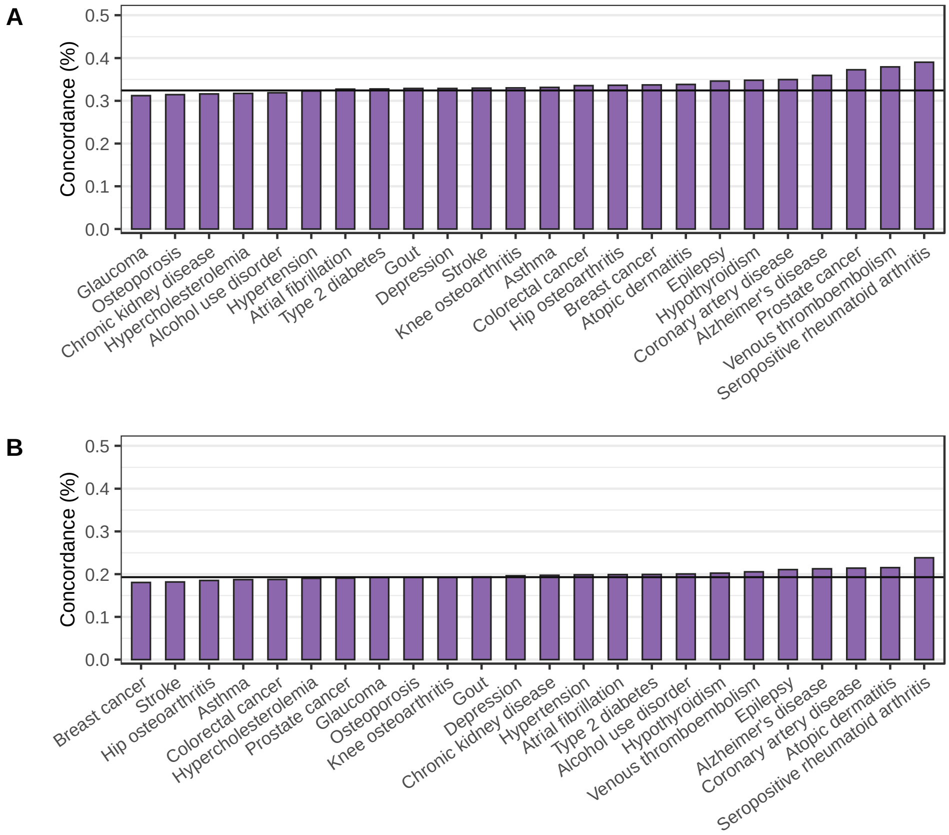

Figure S7. Polygenic risk score (PRS) concordance. Concordance of a high PRS (defined as top 10% of the distribution) among first-degree relatives (**panel A**) and among second-degree relatives (**panel B**). The horizontal lines denote the theoretically derived concordance estimates of 32.4% (**panel A**) and 19.3% (**panel B**) calculated based on reference 47 for first-degree relatives using a high PRS defined as the top 10% of the distribution. Sample sizes: panel A total N = 39,444, N = 15,281 for breast cancer, N = 9,473 for prostate cancer; panel B second -degree family history total N = 47,154, N = 18,973 for breast cancer, N = 12,355 for prostate cancer.

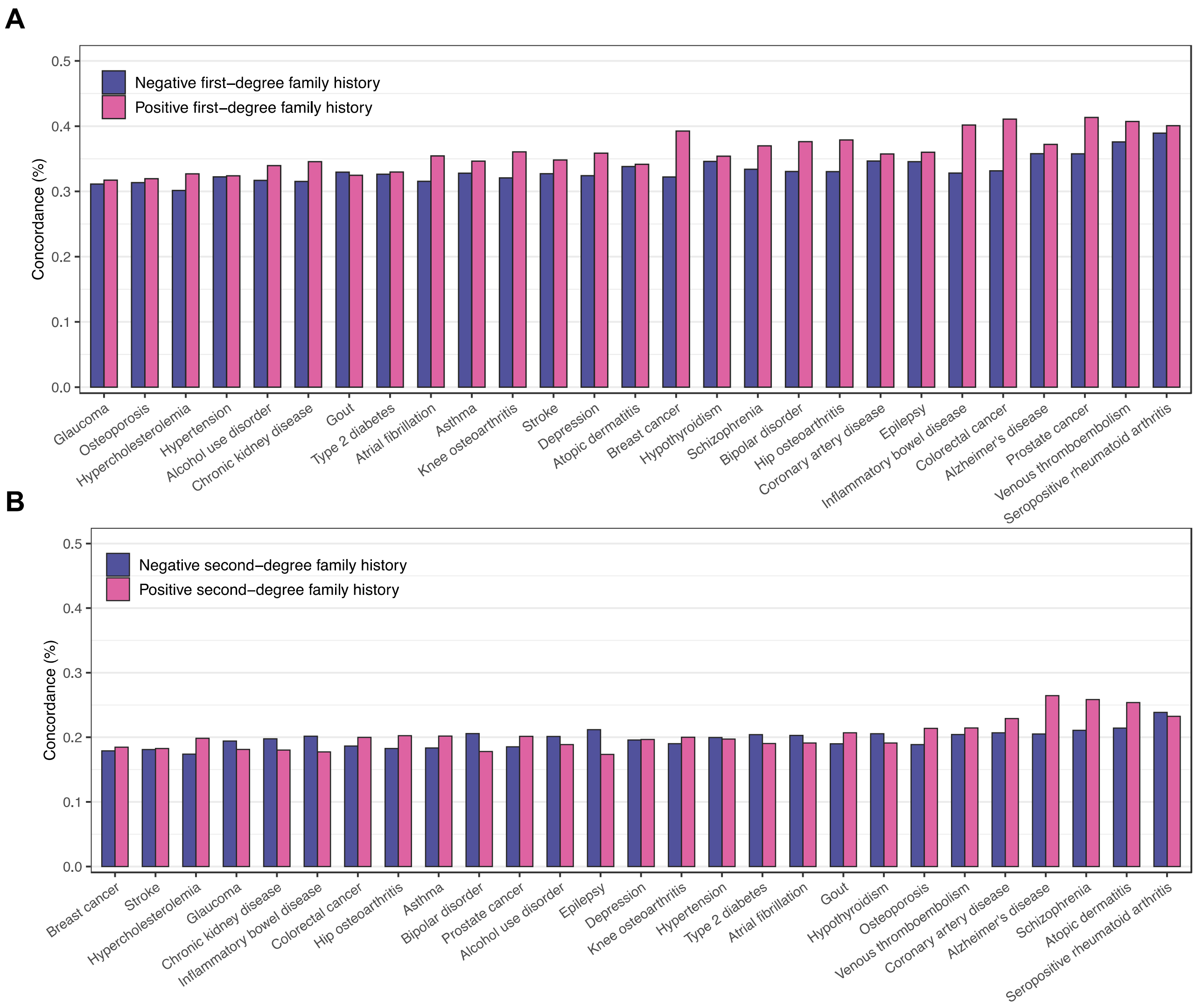

Figure S8. Polygenic risk score (PRS) concordance by family history status. Concordance of a high PRS (defined as top 10% of the distribution) among first-degree relatives (**panel A**) and among second-degree relatives (**panel B**) stratifying by the respective family history status. Sample sizes: panel A total N = 39,444, N = 15,281 for breast cancer, N = 9,473 for prostate cancer; panel B second -degree family history total N = 47,154, N = 18,973 for breast cancer, N = 12,355 for prostate cancer.

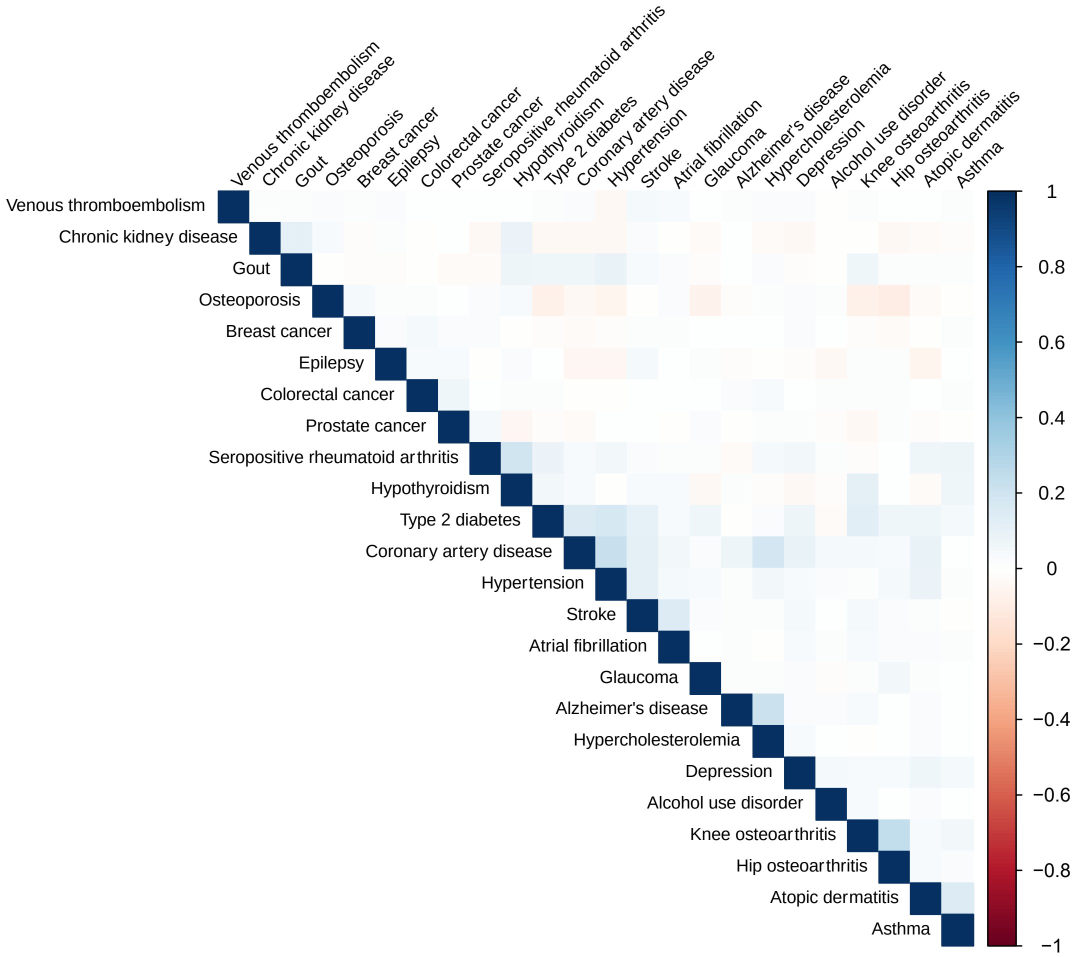

Figure S9. Polygenic risk scores (PRS) correlations .Pearson correlation of the 24 disease-specific PRSs assessed on the continuous scale.

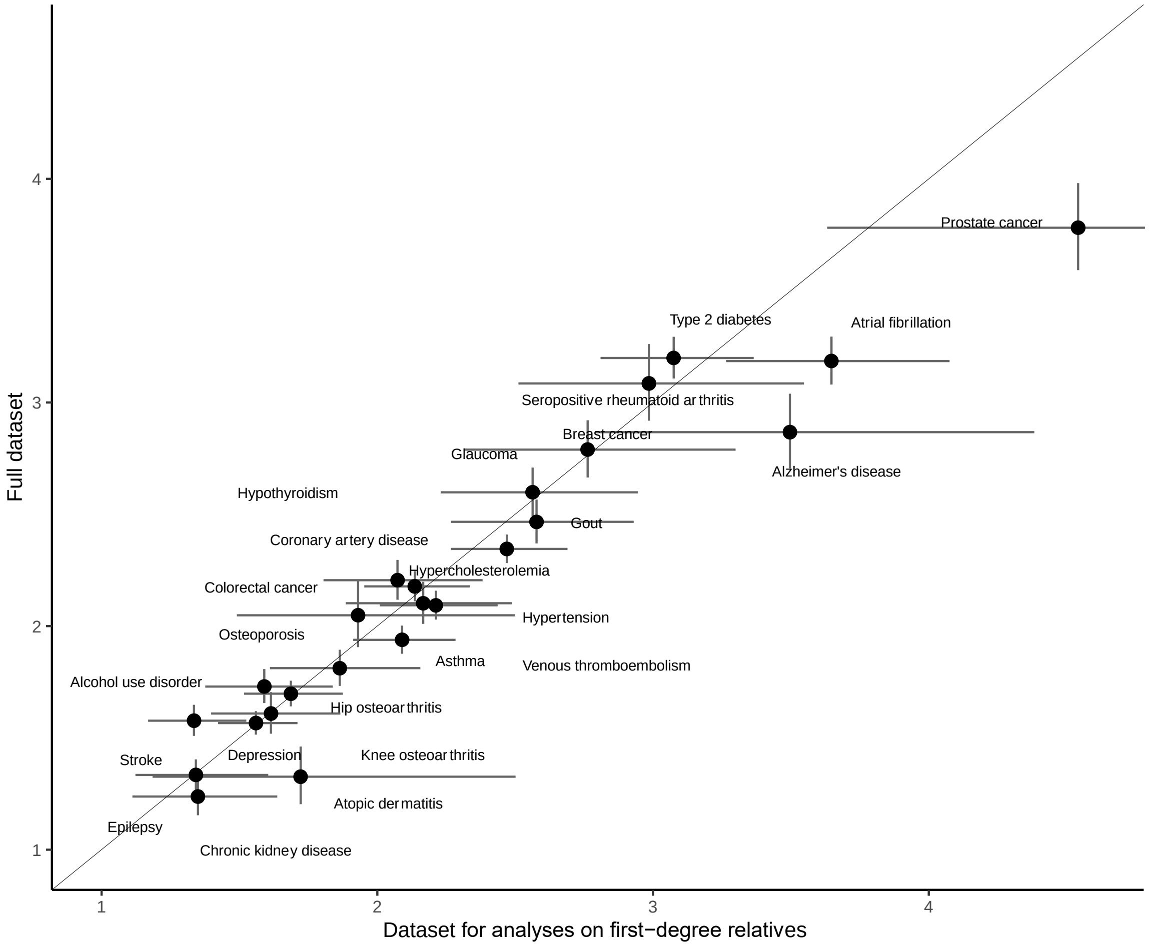

Figure S10. Effect size comparison for the full FinnGen dataset and the dataset used for analyses on first-degree relatives. Effect sizes for a high polygenic risk score (PRS; defined as top 10% of the distribution), comparing individuals in the full data (N = 306,418) on the y axis and the dataset used for analyses on first-degree relatives (N = 39,444) on the x axis. The PRS effect sizes were similar in both.

Supplemental Material and Methods

Identification of individuals with diseases included harmonization of diagnoses according to different revisions of International Statistical Classification of Diseases (ICD-8/9/10) (**Table S3**). Registries used include the hospital discharge registry (available from 1968-), the Finnish Cancer Registry (1953-), causes of death registry (1964-), and the medication reimbursement and medication purchases registries (1964- and 1995-), both administered by the Social Insurance Institute of Finland. Age at disease onset was defined as the age at the first healthcare contact with the disease. Age at death from the causes of death registry as recorded on the death certificate and checked by Statistics Finland. Parental cause of death (FH_P) was defined as at least one parent having the disease as a cause of death. Prostate cancer was studied only in men and breast cancer only in women.

Genotyping and imputation

FinnGen samples were genotyped with Illumina and Affymetrix arrays (Illumina Inc., San Diego, and Thermo Fisher Scientific, Santa Clara, CA, USA), and genotype calls were made with the GenCall or zCall (for Illumina) and the AxiomGT1 algorithm for Affymetrix data. Individuals with ambiguous gender (markers on the X chromosome not matching the sex denoted by the personal identification number), high genotype missingness (>5%), excess heterozygosity (+-4SD) and non-Finnish ancestry were excluded, as well as all variants with high missingness (>2%), low Hardy–Weinberg equilibrium p-value (<1e-6) and minor allele count (MAC < 3). Array data pre-phasing was carried out with Eagle 2.3.5[1] with the number of conditioning haplotypes set to 20,000. Genotype imputation was done using the population-specific SISu v3 imputation reference with 3,775 high-coverage (25-30x) whole-genome sequences in Finns, described in detail at https://doi.org/10.17504/protocols.io.xbgfijw.

Polygenic risk scores

Details on the GWASs used as the input for the PRSs are available in **Table S2**. Using these as priors, we applied the PRS-CS-auto algorithm to infer posterior effect sizes for the variants for PRS calculation. PRS-CS-auto learns the model's global scaling parameter ϕ from the data, performing well with large datasets.[2] The PRS-CS pipeline in FinnGen is described at https://github.com/FINNGEN/CS-PRS-pipeline. The PRSs for autosomes were calculated using PLINK v2.00a2.3LM, by calculating the weighted sum of risk alleles for each variant with the parameter *--score* applied on a genotype file (all chromosomes combined) which had been filtered to 1,194,526 HapMap3 variants, as recommended for PRS-CS. The mean number of variants included in the PRSs was 1,059,217. We observed very little correlation between the PRSs (**Figure S9**). To avoid overfitting of effects, the individuals potentially overlapping with the GWASs were excluded from all analyses based on genotyping array and cohort information (**Table S1, Table S2**). As we are unable to identify the exact individuals overlapping, we chose to use this conservative exclusion approach.

Inferring relatedness

To define first-degree family history (FH_{1st}), we inferred first-degree relatedness from genotypes based on 173,907 independent linkage disequilibrium (LD)-pruned common variants 9 (PLINK parameters --snps-only --chr 1-22 --max-alleles 2 --maf 0.01 --indep-pairwise 500.0 50.0 0.15). The LD pruning was done using variants with INFO >0.9. Using KING v2.2.4[3], pairs of first-degree relatives were identified with a kinship coefficient between 0.177 and 0.354, and pairs of second-degree relatives with a kinship coefficient between 0.0884 and 0.177. To avoid individuals appearing multiple times on either side of the regression equation which would violate the assumption of independence of observations, we performed several steps of random exclusions and exclusions of cohorts predominantly family-based ascertainment (**Figure S1**). We inferred the risk for the individual born later and the individual born earlier was chosen as the relative (random choice for dizygotic twins). The PRS effect sizes were similar in the full data and in those with a first-degree relative in the dataset (**Figure S10**). In a previous study,[4] we performed a comparison of self-reported first-degree family history and PRSs for coronary artery disease (CAD) and type 2 diabetes (T2D) in the Finnish FINRISK cohort. The prevalence of first-degree family history was slightly higher in FINRISK (22.2% for CAD and 25.8% in T2D) than the prevalences for FH_{1st} observed here (15.1% for CAD and 18.6% for T2D; **Table S4**), but the dynamics of family history and PRS were highly similar.

Parental causes of death

Parental causes of death (FH_P) was available for participants regardless of whether their parents are included in FinnGen. FH_P was obtained through the Death Registry, which has nationwide coverage. Information on the parents' sex, age at death, and causes of death (immediate, contributing, and underlying causes of death) was available for FinnGen participants. We studied FH_P to obtain a robust and complementary source of data

that does not have the same limitations as the approach used for FH_{1st} and $FH_{2nd,}$ which required that at least one relative is included in FinnGen.

Ethics statement

Individuals and controls in FinnGen provided informed consent for biobank research, based on the Finnish Biobank Act. Alternatively, separate research cohorts, collected prior the Finnish Biobank Act came into effect (in September 2013) and start of FinnGen (August 2017), were collected based on study-specific consents and later transferred to the Finnish biobanks after approval by Fimea (Finnish Medicines Agency), the National Supervisory Authority for Welfare and Health. Recruitment protocols followed the biobank protocols approved by Fimea. The Coordinating Ethics Committee of the Hospital District of Helsinki and Uusimaa (HUS) statement number for the FinnGen study is Nr HUS/990/2017.

The FinnGen study is approved by Finnish Institute for Health and Welfare (permit numbers: THL/2031/6.02.00/2017, THL/1101/5.05.00/2017, THL/341/6.02.00/2018, THL/2222/6.02.00/ 2018, THL/283/6.02.00/2019, THL/1721/5.05.00/2019, THL/1524/5.05.00/2020, and THL/2364/ 14.02/2020), Digital and population data service agency (permit numbers: VRK43431/2017-3, VRK/6909/2018-3, VRK/4415/2019-3), the Social Insurance Institution (permit numbers: KELA 58/522/2017, KELA 131/522/2018, KELA 70/522/2019, KELA 98/522/2019, KELA 138/522/2019, KELA 2/522/2020, KELA 16/522/2020, Findata THL/2364/14.02/2020 and Statistics Finland (permit numbers: TK-53-1041-17 and TK/143/07.03.00/2020 (earlier TK-53-90-20).

The Biobank Access Decisions for FinnGen samples and data utilized in FinnGen Data Freeze 7 include: THL Biobank BB2017_55, BB2017_111, BB2018_19, BB_2018_34, BB_2018_67, BB2018_71, BB2019_7, BB2019_8, BB2019_26, BB2020_1, Finnish Red Cross Blood Service Biobank 7.12.2017, Helsinki Biobank HUS/359/2017, Auria Biobank AB17-5154 and amendment #1 (August 17 2020), Biobank Borealis of Northern Finland_2017_1013, Biobank of Eastern Finland 1186/2018 and amendment 22 § /2020, Finnish Clinical Biobank Tampere MH0004 and amendments (21.02.2020 & 06.10.2020), Central Finland Biobank 1-2017, and Terveystalo Biobank STB 2018001.

FinnGen acknowledgements

We would like to thank Mervi Aavikko and Risto Kajanne for management assistance. The FinnGen project is funded by two grants from Business Finland (HUS 4685/31/2016 and UH 4386/31/2016) and the following industry partners: AbbVie Inc., AstraZeneca UK Ltd, Biogen MA Inc., Bristol Myers Squibb, Genentech Inc., Merck Sharp & Dohme Corp, Pfizer Inc., GlaxoSmithKline Intellectual Property Development Ltd., Sanofi US Services Inc., Maze Therapeutics Inc., Janssen Biotech Inc, and Novartis Pharma AG. Following biobanks are acknowledged for delivering biobank samples to FinnGen: Auria Biobank (www.auria.fi/biopankki), THL Biobank (www.thl.fi/biobank), Helsinki Biobank (www.helsinginbiopankki.fi), Biobank Borealis of Northern Finland (https://www.ppshp.fi/Tutkimus-ja-opetus/Biopankki/Pages/Biobank-Borealis-briefly-in-English.aspx), Finnish Clinical Biobank Tampere (www.tays.fi/en-US/Research_and_development/Finnish_Clinical_Biobank_Tampere), Biobank of Eastern Finland (www.ita-suomenbiopankki.fi/en), Central Finland Biobank (www.ksshp.fi/fi-FI/Potilaalle/Biopankki), Finnish Red Cross Blood Service Biobank (www.veripalvelu.fi/verenluovutus/biopankkitoiminta) and Terveystalo Biobank (www.terveystalo.com/fi/Yritystietoa/Terveystalo-Biopankki/Biopankki/). All Finnish Biobanks are members of BBMRI.fi infrastructure (www.bbmri.fi) and FINBB biobank cooperative (https://finbb.fi/) is the coordinator of the BBMRI-ERIC operations in Finland covering all Finnish biobanks.

Supplemental references

1. Loh, P.R., Danecek, P., Palamara, P.F., Fuchsberger, C., Reshef, Y.A., Finucane, H.K., Schoenherr, S., Forer, L., McCarthy, S., Abecasis, G.R., et al. (2016). Reference-based phasing using the Haplotype Reference Consortium panel. Nat. Genet. *48*, 1443-1448.
2. Ge, T., Chen, C.Y., Ni, Y., Feng, Y.A., and Smoller, J.W. (2019). Polygenic prediction via Bayesian regression and continuous shrinkage priors. Nat Commun *10*, 1776.
3. Manichaikul, A., Mychaleckyj, J.C., Rich, S.S., Daly, K., Sale, M., and Chen, W.M. (2010). Robust relationship inference in genome-wide association studies. Bioinformatics *26*, 2867-2873.
4. Mars, N., Koskela, J.T., Ripatti, P., Kiiskinen, T.T.J., Havulinna, A.S., Lindbohm, J.V., Ahola-Olli, A., Kurki, M., Karjalainen, J., Palta, P., et al. (2020). Polygenic and clinical risk scores and their impact on age at onset and prediction of cardiometabolic diseases and common cancers. Nat. Med. *26*, 549-557.
